# Diagnostic Fragmentations of Animal and Fungal Sterols/Stanols Obtained by APCI–Tandem Mass Spectrometry: A Route Towards Unknown Free Sterol Identification

**DOI:** 10.3390/metabo15100674

**Published:** 2025-10-16

**Authors:** Valeria Cinquepalmi, Ilario Losito, Andrea Castellaneta, Cosima Damiana Calvano, Tommaso R. I. Cataldi

**Affiliations:** 1 Dipartimento di Chimica, Università degli Studi di Bari “Aldo Moro”, Via E. Orabona 4, 70126 Bari, Italy; valeria.cinquepalmi@uniba.it (V.C.); andrea.castellaneta@uniba.it (A.C.); cosimadamiana.calvano@uniba.it (C.D.C.); tommaso.cataldi@uniba.it (T.R.I.C.); 2 Centro Interdipartimentale SMART, Università degli Studi di Bari “Aldo Moro”, Via E. Orabona 4, 70126 Bari, Italy

**Keywords:** animal/fungal sterols and stanols, atmospheric pressure chemical ionization, high resolution tandem mass spectrometry, higher-collisional-energy dissociation, fragmentation pathways

## Abstract

**Background/Objectives**: Animal and fungal sterols and stanols exhibit remarkable structural diversity, driven by variations in the number and position of C=C bonds within the steroidal tetracyclic core and side chain, along with diverse branching patterns of the latter. Similarly to phytosterols, these metabolites produce highly complex tandem mass spectra, whose interpretation has so far been limited. To address this gap, the fragmentation behavior of selected animal/fungal sterols and stanols was studied in this paper. **Methods**: Higher-Collisional-energy Dissociation–High-resolution tandem mass spectrometry (HCD-HRMS/MS) of protonated/dehydrated species generated via atmospheric pressure chemical ionization (APCI) was performed on structurally diverse compounds, including lathosterol, desmosterol, zymosterol, lanosterol, ergosterol, chalinasterol, and the stanols coprostanol and cholestanol. **Results**: Structurally diagnostic product ions originating from the side chains were unveiled, shedding light on the intramolecular migration of positive charge from the initial ionization site at C3 to alternative stable sites across the molecular structure, which is a typical mechanism also noted in cholesterol and phytosterols. In addition, characteristic fragmentation patterns related to the steroidal backbone were found and discussed for Δ^7^, Δ^5,7^ and Δ^8^-sterols, and a novel elucidation of the fragmentation behavior of 4,4-dimethyl-Δ^8^-sterols, based on lanosterol as a model compound, was achieved. The relative intensities of diagnostic product ions allowed a correlation with specific structural motifs, and “cholesterol-like” and “stigmasterol-like” fragmentations pathways were recognized. These findings were integrated with prior data on cholesterol and plant sterol fragmentation acquired under identical analytical conditions. Moreover, as a proof of its relevance for novel sterol identification, MS/MS-related information was successfully applied to the identification of a positional isomer (Δ^7^) of zymosterol in baker’s yeast extract, along with typical fungal major sterols. **Conclusions**: The comprehensive archive of sterol/stanol fragmentations obtained by APCI-HCD-MS/MS might prove very useful for the future characterization of novel sterol/stanol species in complex matrices.

## 1. Introduction

**Figure 1:**
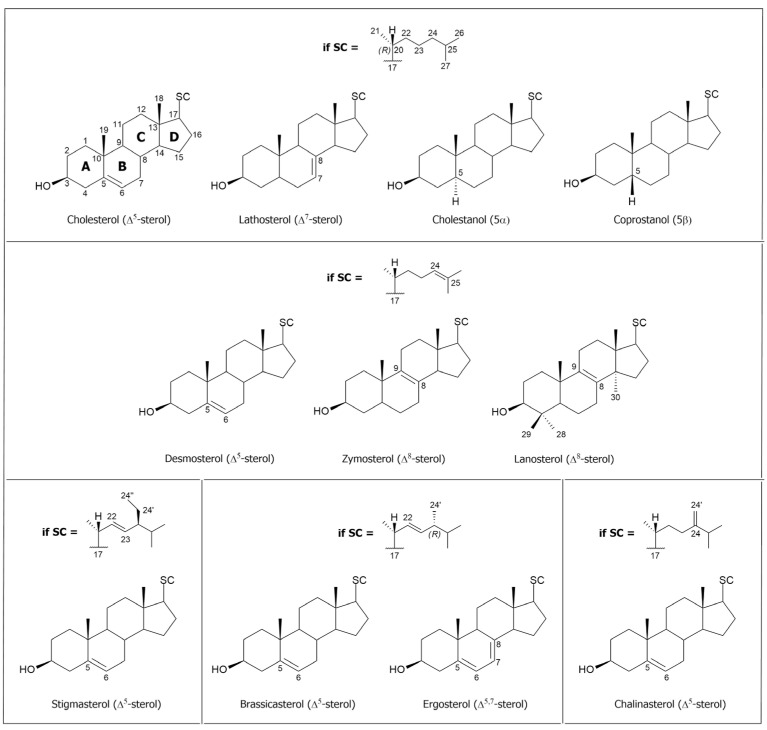
Molecular structures of the animal and fungal sterols analyzed in this study, alongside cholesterol and the phytosterols stigmasterol and brassicasterol, which are included for comparative purposes (see text for additional details). Compounds are grouped based on the side chain (SC) structures. Atom numbering follows the IUPAC guidelines established in 1989. The stereochemical configurations at C20 and C24 are indicated where relevant: R configuration at C20 for cholesterol and related sterols and R configuration at C24 for brassicasterol and ergosterol.

Despite their relatively low abundance, sterols play a crucial role in maintaining the structural integrity and regulating the fluidity and permeability of the lipid bilayer in eukaryotic plasma membranes [1,2,3,4]. From a biosynthetic perspective, sterols originate from the isoprene pathway and are derived through the oxygenation and cyclisation of squalene [1,5,6]. These transformations yield their characteristic tetracyclic structure, a perhydro-1,2-cyclopentanophenanthrene ring system, bearing a hydroxyl group at C3, which imparts them an amphipathic nature [5,7]. The four rings of the steroidal backbone, labelled A, B, C, and D (Figure 1), adopt an all-*trans-anti* configuration, resulting in a nearly planar molecular conformation [3,8]. Atom numbering follows the IUPAC recommendations issued in 1989 [9], wherein C17 is the carbon atom linked to the side chain (SC), another key structural feature of sterols [3,8]. Depending on their biosynthetic origin, sterols may possess none (4-desmethyl-sterols), one (4-methyl-sterols), or two (4,4-dimethyl-sterols) methyl groups at C4. Additionally, a methyl group may be present at C14, as observed in lanosterol (Figure 1). Sterols are further classified based on the number and position of double bonds on the B ring, giving rise to Δ^5^-, Δ^7^-, Δ^5,7^-, and Δ^8^-sterols. Stanols, in contrast, are their fully saturated counterparts [3,8].

The specific sterol and stanol profiles are strongly organism-dependent, as their biosynthesis follows phylum-specific pathways [5]. In plants, for example, sterol methyltransferases (SMTs) introduce characteristic alkyl branches at C24, producing major plant sterols, such as β-sitosterol and campesterol [10,11]. In contrast, both animal and fungal sterols are biosynthesized from a common precursor, lanosterol, which features a C=C double bond between C24 and C25 (Figure 1). From lanosterol, two parallel pathways diverge: the Bloch pathway, which leads to sterols such as desmosterol and zymosterol, which retain lanosterol’s side chain but differ in B-ring unsaturation [5,7], and the Kandutsch–Russell pathway, which gives rise to sterols with saturated, unbranched side chains, such as lathosterol, a Δ^7^-positional isomer of cholesterol [5,6,12,13]. Notably, both lathosterol and desmosterol serve as biosynthetic precursors to cholesterol, the most abundant sterol in animal cells [2]. The sterols discussed so far are critical to human health, and imbalances in their levels have been linked to disorders of the nervous and skeletal systems [12]. Among cholesterol metabolites, cholestanol and coprostanol are epimers with saturated B rings and differ only in the stereochemistry of the hydrogen at C5 (α or β, respectively; Figure 1), yielding either a planar or a bent A ring conformation. Both metabolites are produced from cholesterol in the digestive tract [14,15,16,17], although cholestanol has also been reported in higher plants [18]. Fungal sterols also originate from lanosterol. In fungi, zymosterol proceeds along a modified Bloch pathway involving several enzymatic transformations, ultimately producing ergosterol, a Δ^5,7^-sterol with a double bond between C22 and C23 and a methyl group at C24 in the R configuration (Figure 1) [4,13]. Ergosterol is the dominant sterol in fungal cells, including yeasts and molds, where it plays a role analogous to cholesterol in animal membranes, supporting membrane integrity and cell proliferation [2,4,19]. Among the sterols illustrated in Figure 1, chalinasterol is a Δ^5^-sterol containing a methylene group at C24. It is a positional isomer of brassicasterol and a biosynthetic precursor of campesterol, both of which are important plant sterols [20]. Also known as 24-methylene-cholesterol or ostreasterol, chalinasterol is considered a “marine sterol” due to its initial identification in mollusks [21] and subsequent detection in algae [22,23,24], though it is also present in various vegetable oils [25].

The structural diversity of sterols poses a significant challenge in terms of analytical characterization. Gas chromatography–mass spectrometry with electron ionization (GC-EI-MS) remains a standard technique for sterol analysis, although it typically requires prior derivatization to enhance volatility and ionization efficiency [26,27,28,29,30,31]. As an alternative, reversed-phase liquid chromatography coupled with soft ionization mass spectrometry techniques such as electrospray ionization (ESI) or atmospheric pressure chemical ionization (APCI) has become increasingly popular [32,33]. APCI, especially in positive ion mode, has shown superior sensitivity for sterol analysis due to the predominant formation of [M+H–H_2_O]^+^ ions [8,32,34,35] and avoids the risk of thermal degradation associated with GC.

Animal sterols and stanols are typically studied in biological matrices like plasma, serum, and feces [31,32,33,34,36,37,38,39], whereas ergosterol serves as a marker of fungal and mold contamination in samples such as soil and yeast [40,41,42]. Nevertheless, these sterols can also be found in plant-derived matrices and algae [24,25,43,44,45]. During chromatographic separation, complex traces with multiple peaks are frequently observed, suggesting the presence of isomeric sterols not corresponding to available standards. In such cases, even high-resolution mass spectrometry (HRMS) fails in providing useful information, and the use of tandem or multistage mass spectrometry (MS/MS or MSⁿ) is required to achieve a deeper structural insight. In recent years, novel approaches for sterol/stanol analysis based on MS/MS have been proposed in the literature, focusing on the all-ion fragmentation technique [46], on fast chromatography [47], or, very recently, on prior derivatization with 3-(chlorosulfonyl)benzoic acid, aiming at detecting sterols as negatively charged ions [48].

As demonstrated in our recent work on cholesterol and major 4-desmethyl plant sterols [49], the combination of high-resolution tandem MS with low-resolution MSⁿ (*n* = 2–4) on [M+H–H_2_O]^+^ ions generated by APCI can reveal diagnostic product ions, which in turn may enable the identification of key structural features, such as side chain unsaturation/branching and variations in the C=C bond location on the steroidal core. The current study applies the same analytical strategy to a broader selection of animal and fungal sterols, exhibiting structural diversity beyond that of cholesterol and plant sterols. The resulting identification of new diagnostic fragment ions, linked to the presence or absence of C=C bonds on ring B and on the side chain and to specific methylation patterns in the steroidal backbone will be discussed in detail, culminating in a comprehensive summary of fragmentation behaviors that may facilitate the structural elucidation of novel sterols from animal, fungal, and plant sources.

## 2. Materials and Methods

### 2.1. Chemicals

LC-MS-grade acetonitrile (ACN), water (H_2_O) and formic acid (FA), adopted for RPLC separations, and HPLC-grade chloroform (CHCl_3_), ethanol (EtOH), methanol (MeOH) and hexane, employed as solvents for the preparation of sterol stock solutions and for the extraction of free sterols from commercial baker’s yeast, were purchased from Merck (Milan, Italy). Standards of cholesterol (cholest-5-en-3β-ol, ≥95% purity), lathosterol (cholest-7-en-3β-ol, ≥95% purity), cholestanol (5α-cholestan-3β-ol, ≥95% purity), coprostanol (5β-cholestan-3β-ol, ≥95% purity), desmosterol (cholest-5,24-dien-3β-ol, ≥98% purity), lanosterol (lanosta-8,24-dien-3β-ol, ≥95% purity) and ergosterol (ergosta-5,7,22E-trien-3β-ol, ≥95% purity) were procured from Cayman Chemical (Ann Arbor, MI, USA). Standards of zymosterol (5α-cholesta-8,24-dien-3β-ol, ≥99% purity) and chalinasterol (24-methylene-cholest-5-en-3β-ol, ≥99% purity) were purchased from Merck (Milan, Italy). Solutions for mass spectrometer calibration under positive or negative polarity conditions were acquired from Thermo Scientific (Waltham, MA, USA).

### 2.2. Standard Solutions Preparation

A stock solution of each standard sterol (1 mg/mL) was prepared in an ethanol/chloroform mixture (2:1 *v*/*v*) and a diluted solution (30 μg/mL) was subsequently obtained in 100% acetonitrile. A comprehensive mix of standards (10 μg/mL for each one) was obtained in 100% acetonitrile through appropriate mixing and dilution of the respective stock solutions.

### 2.3. Extraction of Free Sterols from Baker’s Yeast

Baker’s yeast, purchased in a local store, was selected as an example of a sterol-containing complex matrix and was subjected to extraction of free sterols following the protocol reported in the study by Castellaneta et al. [44], with appropriate modifications. In detail, 50 mg of baker’s yeast was weighed, 1 mL of MeOH was added and the obtained suspension was sonicated in a water bath for 15 min at 30 °C. Then, 3.5 mL of H_2_O and 3.75 mL of hexane were added to extract free sterols. The mixture was vortexed for 2 min and centrifuged at 5000 rpm for 10 min. Following complete phase separation, the supernatant was collected, and the extraction was repeated to increase the recovery of free sterols. The two supernatants were then joined and dried under a gentle flow of nitrogen. The dry residue was then dissolved in 500 μL of a MeOH/CHCl_3_ solution (2:1 *v*/*v*) and diluted by a factor of 1:1 (*v*/*v*) with ACN before RPLC-APCI(+)-FTMS analysis.

### 2.4. RPLC-APCI-MS^n^ Instrumentation and Operating Conditions

APCI-MS^n^ (*n* = 1–3) analyses of sterols/stanols were performed, according to the case, with one of two platforms including an Ultimate 3000 HPLC system coupled either with a Q Exactive high-resolution quadrupole–Orbitrap Fourier-transform mass spectrometer or with a Velos Pro linear ion trap mass spectrometer (ThermoFisher, West Palm Beach, CA, USA) through an APCI source. A C18 Ascentis Express HPLC column (150 × 2.1 mm i.d., particle size 2.7 μm) (Supelco, Bellefonte, PA, USA) was used for preliminary chromatographic tests aimed at assessing the possibility of separating isomeric sterols under either isocratic or binary elution gradient conditions similar to those adopted for cholesterol and phytosterols in our previous work [49]. In the first case, 100% ACN with 0.1% (*v*/*v*) FA was used as a mobile phase, working at a flow rate of 0.200 mL/min for 40 min. In the second case, the following elution gradient based on H_2_O as phase A and ACN as phase B, both containing 0.1% (*v*/*v*) of FA, was adopted for sterol separation: 0–40 min, linear increase in B from 90% to 100%; 40–50 min, isocratic at 100% B; 50–52 min, linear decrease in B from 100% to 90%; and 52−60 min, isocratic at 90% B (column reconditioning). In both cases, the column temperature was maintained at 30 °C and 5 μL sample volumes were injected. The parameters of the APCI interface and of the ion optics of the Q Exactive spectrometer were set as follows: sheath gas flow rate 40 a.u.; auxiliary gas flow rate 10 a.u.; capillary temperature 300 °C; S-lens RF Level 55 a.u.; and vaporizer temperature 250 °C. Full-scan FTMS acquisitions in positive ion mode were obtained in a 250–500 *m*/*z* interval, working with a 140,000 resolving power at *m*/*z* 200; the Automatic Gain Control (AGC) level was set at 1 × 10^6^ and maximum injection time was 100 ms. HCD-FTMS/MS acquisitions were performed by the Q-Exactive spectrometer at a 35000 resolving power, selecting precursor ions in a 1.0 *m*/*z* unit-wide isolation window, using a normalized collisional energy (NCE) of 30 units and acquiring the tandem mass spectrum in a 50−500 *m*/*z* interval; the AGC level was set at 2 × 10^5^ and maximum injection time was 100 ms. The spectrometer was calibrated every two days by infusing, at a 30 μL/min flow rate, calibration solutions for positive or negative polarity acquisitions provided by the instrument manufacturer. As a result, a mass accuracy better than 5 ppm was achieved, with the exception of product ions obtained by HCD-FTMS/MS with *m*/*z* values lower than 100, for which an accuracy better than 16 ppm was, in any case, achieved (*vide infra*).

APCI–direct infusion analyses (APCI-DIA) of individual standards solutions (30 μg/mL) were performed using the Q Exactive spectrometer, under the same conditions described before, and, in specific cases, the Velos Pro mass spectrometer. Specifically, APCI-DIA-HCD-FTMS/MS spectra were acquired for each sterol (*vide infra*) using the Q Exactive spectrometer, whereas APCI-DIA–collisionally induced dissociation (CID)–MS^3^ experiments were performed with the Velos Pro spectrometer on specific precursor ions previously detected in CID-MS/MS spectra obtained with the same spectrometer in order to elucidate some fragmentation patterns observed under HCD-FTMS/MS conditions. The main operating parameters of the Velos Pro APCI interface and ion optics were set as follows: sheath gas flow rate 40 a.u.; auxiliary gas flow rate 10 a.u.; capillary temperature 300 °C; S-lens RF Level 60 a.u.; and vaporizer temperature 250 °C. A 1.0 *m*/*z* unit-wide isolation window was adopted for the isolation of the precursor ion and a normalized collisional energy (NCE) of 25 units was selected for fragmentation in the case of CID-MS/MS and CID-MS^3^ acquisitions performed using the linear ion trap spectrometer; the AGC level was set at 2 × 10^3^ and the maximum injection time was 100 ms.

## 3. Results and Discussion

### 3.1. RPLC-APCI(+)-FTMS Analysis of Standard Sterols/Stanols: Chromatographic Considerations

**Figure 2:**
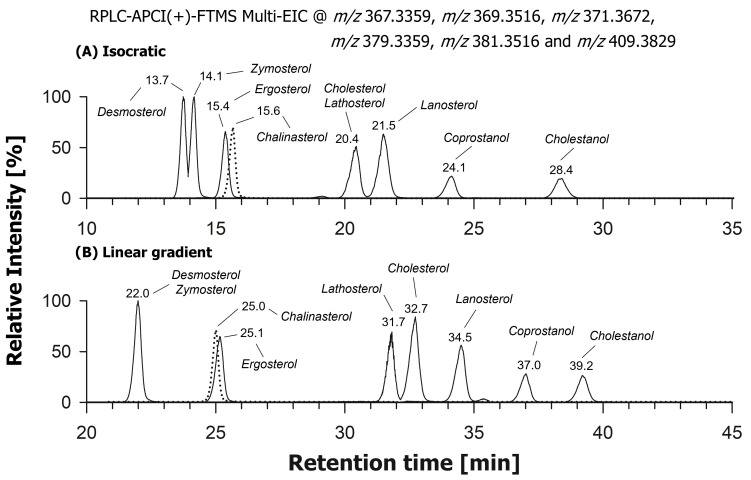
Multiple-extracted ion current (EIC) chromatograms obtained from the RPLC-APCI(+)-FTMS analysis, performed using the Q Exactive mass spectrometer, of the standard sterols and stanols examined in this study, along with cholesterol, based on: (**A**) isocratic elution and (**B**) linear gradient elution. Ion currents were extracted using the exact monoisotopic *m*/*z* values of the [M+H–H_2_O]^+^ ions of the compounds: *m*/*z* 367.3359 for desmosterol and zymosterol; *m*/*z* 379.3359 for ergosterol; *m*/*z* 381.3516 for chalinasterol (dashed line); *m*/*z* 369.3516 for cholesterol and lathosterol; *m*/*z* 409.3829 for lanosterol; and *m*/*z* 371.3672 for coprostanol and cholestanol. The chromatograms illustrate the retention profiles and resolution achieved for structurally related sterols and stanols under both elution conditions.

Preliminary RPLC separations were conducted on the mixture of animal and fungal sterols and stanols of interest, with the addition of cholesterol, to assess their chromatographic behavior, particularly the resolution of isomeric species, and to gain a deeper insight into how sterol molecular structure influences retention. The findings complement results obtained in a previous study on cholesterol and phytosterols [49] based on the same chromatographic conditions. In that case, a binary gradient elution program using water and acetonitrile containing 0.1% formic acid was developed to separate Δ^7^-avenasterol from its Δ^5^ counterpart, isofucosterol. Applying the same gradient program, along with an isocratic elution using 100% acetonitrile, to the current sterol/stanol mixture produced the multiple-extracted ion current (EIC) chromatograms shown in Figure 2. These chromatograms were targeted at the exact monoisotopic *m*/*z* values of the sterol/stanol respective [M+H–H_2_O]^+^ ions. As expected, isocratic elution yielded a faster separation, due to the higher elution strength of the mobile phase. The elution order observed under isocratic conditions generally mirrored that of the gradient method, with a few notable exceptions.

Stanols, which lack unsaturations, were consistently eluted last and were well resolved under both conditions, highlighting the significant impact of the steroidal backbone stereochemistry on the retention behavior on C18 stationary phases. In particular, coprostanol, in which the β-configuration at C5 imposes a *cis* junction between the A and B rings, eluted earlier than cholestanol, for which the canonical *trans*-planar structure is preserved [17]. This behavior is reminiscent of that observed, under RPLC conditions, with unsaturated fatty acids, where a *cis* C=C bond introduces a kink that weakens the interactions with the C18 chains of the stationary phase, resulting in earlier elution than the *trans* counterpart [50]. Lanosterol’s retention behavior further illustrates the complex role played by the backbone structure. Despite the polarity induced by the occurrence of a C=C bond between C24 and C25 (see Figure 1), a feature previously found to significantly impact retention [49], lanosterol was consistently eluted after cholesterol, which has a saturated side chain. This result might be tentatively explained by the presence of two methyl groups at C4 in lanosterol, which form a localized hydrophobic domain near the hydroxyl group. This domain likely reduces the effect of OH polarity, enhancing the sterol affinity for the C18 phase.

The first critical resolution challenge, moving from longer to shorter retention times, was the separation of cholesterol from its isomer lathosterol. These sterols differ only in the position of a single double bond on the B ring (Figure 1). Like Δ^5^/Δ^7^-avenasterols [49], these isomers could not be separated under isocratic conditions. However, gradient elution (see Figure 2B) allowed lathosterol (a Δ^7^ sterol, thus having its C=C bond closer to the side chain) to elute earlier than cholesterol (a Δ^5^ sterol). This trend agrees with previous findings by McDonald et al. [37] and Honda et al. [33,34], suggesting that a slow, gradient-based increase in acetonitrile content is more effective in separating sterols based on double bond position within the steroidal backbone. Unexpectedly, however, isocratic elution outperformed gradient elution in resolving the remaining four sterols under study. As shown in Figure 2A, ergosterol and chalinasterol were consistently eluted before the cholesterol/lathosterol pair, as expected due to the presence of a side chain double bond. On the other hand, the complex interplay between the different position of this C=C bond and the different levels of unsaturation on ring B, with ergosterol being a Δ^5,7^ sterol and chalinasterol a Δ^5^ one (see Figure 1), resulted in a very similar retention for the two compounds. Surprisingly, isocratic elution slightly improved their resolution, due to the 0.1 min increase in the retention time difference, compared to gradient elution (Figure 2A). This outcome is emphasized by values of resolution and selectivity evaluated for all the species’ couples under both chromatographic setups, which are summarized in Table S1. In any case, the distinct *m*/*z* values, due to ergosterol’s additional double bond compared to chalinasterol, allowed a clear identification of the two compounds via the respective EIC chromatograms. Further support for the influence of the side chain C=C bond position on retention was obtained when comparing chalinasterol with brassicasterol, which shares the same backbone and was analyzed during our previous investigation [49]. Chalinasterol, including a C=C bond between C24 and C24′ (Figure 1), eluted far earlier than brassicasterol, whose double bond is closer to the backbone (between C22 and C23). Indeed, under the same gradient conditions, brassicasterol showed a retention time of 31.7 min [49], over 6.7 min longer than chalinasterol.

An advantage for isocratic elution was also observed with isomeric species desmosterol and zymosterol (Figure 2). Both sterols feature a C=C bond between C24 and C25, significantly reducing their retention compared to all others, including lanosterol, which shares their side chain but has additional methylations at C4 and C14. The two sterols could be slightly separated only under isocratic conditions, which would thus be crucial for identifying them in real samples, given the identical *m*/*z* value for the respective ions. Interestingly, desmosterol (the Δ^5^ isomer) eluted before zymosterol (the Δ^8^ isomer), in contrast to trends observed under gradient elution conditions for Δ^5^/Δ^7^ couples, like the two avenasterols [49] and cholesterol/lathosterol. One possible explanation for this finding likely lies in the accessibility of the C8–C9 double bond in zymosterol, which may be hindered by nearby axial methyl groups on C18 and C19. This steric hindrance could reduce the interaction with the C18 stationary phase, lessening the retention decrease typically associated with double bonds near the side chain. Notably, McDonald et al. [37] and Honda et al. [34] achieved desmosterol–zymosterol separation using RPLC with a water/methanol-based mobile phase but failed to separate cholesterol from lathosterol. In contrast, Skubic et al. [31] achieved excellent separation of animal sterols, including Δ^7^ and Δ^8^ isomers, using two pentafluorophenyl columns in series and a methanol–isopropanol mobile phase, observing shorter retention for Δ^7^ and Δ^8^ isomers relative to Δ^5^ analogues.

Taken together with previous findings on cholesterol and major phytosterols obtained under the same chromatographic conditions [49], these results indicate that relationships between retention time and structural features exist for several sterols. The side chain composition seems to be the most influential structural feature in terms of retention, at least on a C18 stationary phase. However, even the number and position of C=C double bonds on the steroidal backbone and the methylation of specific positions on the latter appear to play a role. This explains why the chromatographic behavior of some critical couples of sterols appears to depend significantly on specific experimental conditions, including not just mobile phase composition or stationary phase type but, in some cases, even the column brand, maybe due to differences in the coverage of support packing particles by octadecyl moieties. A detailed modelling of the sterol/stanol chromatographic behavior might provide a deeper explanation for the findings reported in this section. Nonetheless, given a certain set of chromatographic conditions, retention time comparisons with known standards might support structural inferences for novel sterols detected in real matrices. Moreover, the combined use of isocratic and gradient elution can be considered particularly effective in distinguishing positional isomers, representing the initial step towards the identification of unknown sterols. As shown in the following sections, fragmentation patterns obtained by HCD-FTMS/MS analysis of standard sterols and stanols offered extensive structure-specific information. When combined with chromatographic data, this information might be very helpful for a comprehensive characterization of sterols in complex samples.

### 3.2. Fragmentation of Δ^7^-Sterols with Saturated and Unbranched Side Chain: Lathosterol

In our recent study on cholesterol and major phytosterols, diagnostic product ions arising from their side chains were evidenced by comparison with the results of the RPLC-APCI(+)-FTMS/MS analysis of isotopically labelled cholesterol and stigmasterol standards [49]. These product ions were best explained by assuming alternative locations of the positive charge initially formed at the C3 position, following water loss from protonated sterols during APCI. The positive charge migration was attributed to intramolecular hydride transfers towards C3 occurring in the gas phase, especially from carbons like C25, C17, or C24 (when alkyl-substituted), which better stabilize the positive charge [49]. Starting from this background, APCI(+)-HCD-FTMS/MS spectra were also systematically acquired for the animal/fungal sterols/stanols considered in this study, using the same NCE value adopted for cholesterol and phytosterols, i.e., 30. The spectra were first acquired under DIA conditions and their intensity profiles were subsequently confirmed by those resulting from spectral averaging under the sterol/stanol chromatographic peaks detected upon RPLC-APCI(+)-HCD-FTMS/MS analysis of standard mixtures, performed using linear gradient elution for all sterols/stanols but desmosterol and zymosterol, since the latter could be at least partially separated only under isocratic conditions (see Figure 2A). In this case the RPLC-APCI(+)-HCD-FTMS/MS spectra for the two sterols were obtained by careful spectral averaging in retention time intervals in which the chromatographic peak overlap was minimal.

Notably, preliminary DIA-APCI(+)-HCD-FTMS/MS acquisitions were performed on all sterols/stanols also considering other NCE values, namely 10, 50 and 70. As evidenced in Figures S1–S3 in the Supplementary Materials, at NCE = 10 the fragmentation yield was very low, as expected. Fragmentation was much more effective in generating low-*m*/*z* product ions when 50 and 70 were adopted as NCE values, thus reducing important information related to higher-*m*/*z* ions. Consequently, the NCE = 30 value, the same as that previously adopted in our study on cholesterol and phytosterols [49], emerged as the one leading to the best distribution of spectral intensity among product ions clusters, including those at higher *m*/*z* values, thus potentially providing the maximum amount of structural information.

**Figure 3:**
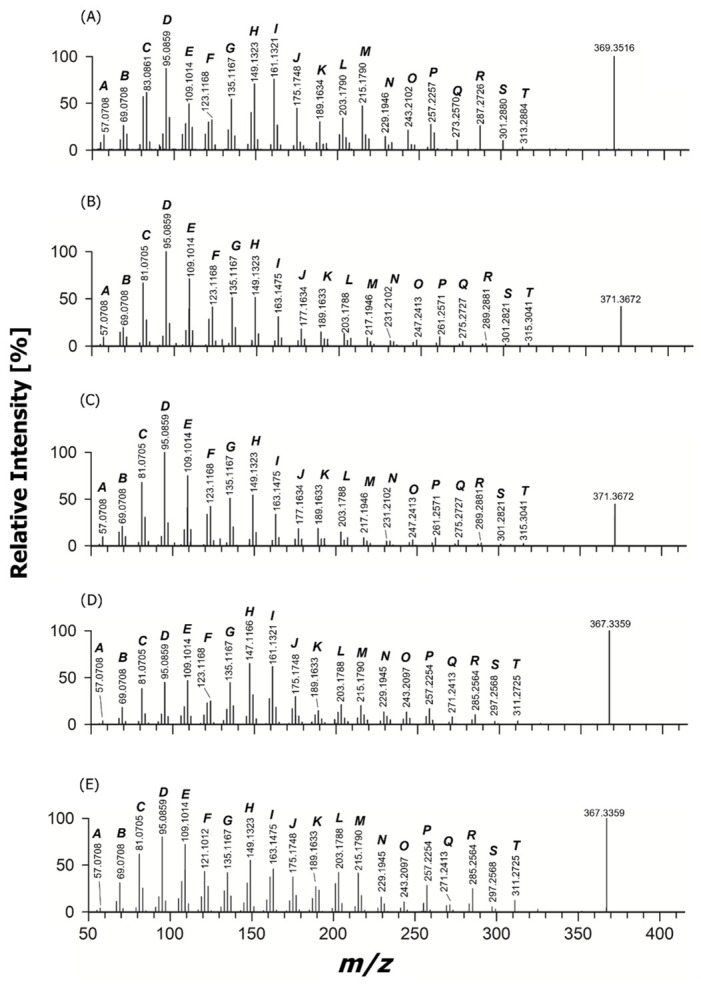
APCI(+)-HCD-FTMS/MS spectra obtained using the Q Exactive mass spectrometer for the [M+H–H_2_O]^+^ ions of selected sterols and stanols analyzed in this study: (**A**) Lathosterol (*m/z* 369.3516); (**B**) Cholestanol (*m/z* 371.3672); (**C**) Coprostanol (*m/z* 371.3672); (**D**) Demosterol (*m/z* 367.3359); (**E**) Zymosterol (*m/z* 367.3359). Peak signals refering to product ions sharing the same number of carbon atoms but differing in the number of H atoms are grouped into clusters, each labeled with a capital letter. For each cluster, the experimental *m*/*z* value of the most abundant ion is usually indicated. The reported spectra were obtained through direct infusion analysis and confirmed by those resulting from spectral averaging under the respective peaks detected in RPLC-APCI(+)-HCD-FTMS/MS chromatograms (see text for details). HCD-FTMS/MS spectra were obtained at NCE = 30, with the HCD cell filled with nitrogen.

The fragmentation pattern of lathosterol, the Δ^7^ analogue of cholesterol, was examined firstly among those of the animal and fungal sterols investigated. The APCI(+)-HCD-FTMS/MS spectrum at NCE = 30 of its [M+H–H_2_O]^+^ ion (exact *m*/*z* for M + 0 isotopologue: 369.3516) is shown in Figure 3A. Product ions with the same carbon count but differing in hydrogen content were grouped into clusters (denoted by capital letters). Table S1 in the Supplementary Materials lists all product ions detected for lathosterol (and for all the other analyzed sterols/stanols), including those corresponding to unlabeled peak signals in Figure 3A. For each ion the experimental *m*/*z* value (rounded to four decimal places), the corresponding molecular formula, inferred considering the best mass accuracy among those related to possible molecular formulas (usually not exceeding 5 ppm, with the exception of *m*/*z* values lower than 96, for which an accuracy up to 16 ppm was considered), and the relative intensity for each sterol, expressed as mean *±* standard deviation calculated from three independent replicates, are reported in the table.

**Figure 4:**
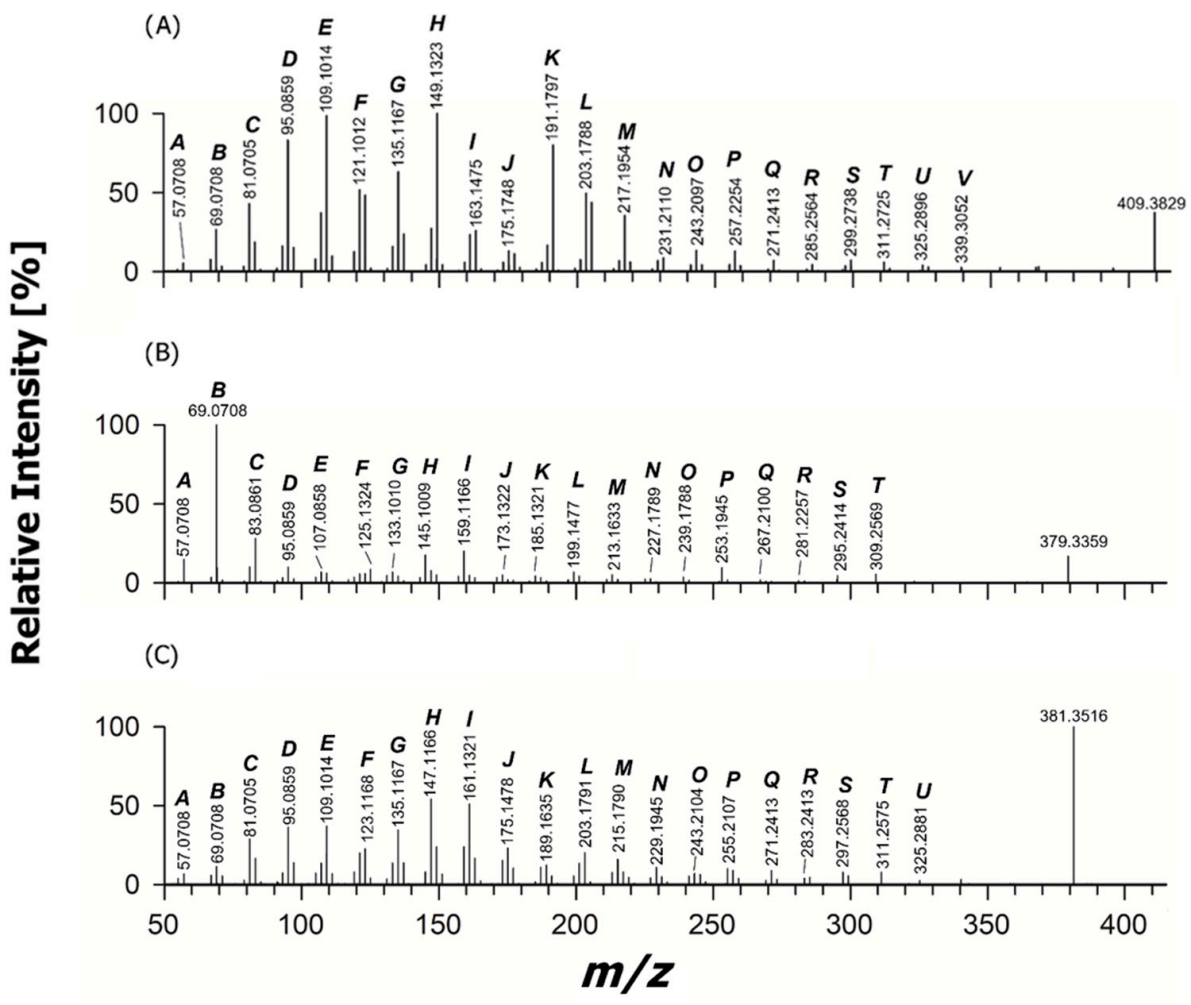
APCI(+)-HCD-FTMS/MS spectra obtained using the Q Exactive mass spectrometer for the [M+H–H_2_O]^+^ ions of selected sterols analyzed in this study: (**A**) lanosterol (*m/z* 409.3829); (**B**) ergosterol (*m/z* 379.3359); and (**C**) chalinasterol (*m/z* 381.3516). Peak signals refering to product ions sharing the same number of carbon atoms but differing in the number of H atoms are grouped into clusters, each labeled with a capital letter. For each cluster, the experimental *m*/*z* value of the most abundant ion is usually indicated. The reported spectra were obtained through direct infusion analysis and confirmed by those resulting from spectral averaging under the respective peaks detected in RPLC-APCI(+)-HCD-FTMS/MS chromatograms (see text for details). HCD-FTMS/MS spectra were obtained at NCE = 30, with the HCD cell filled with nitrogen.

**Scheme 1:**
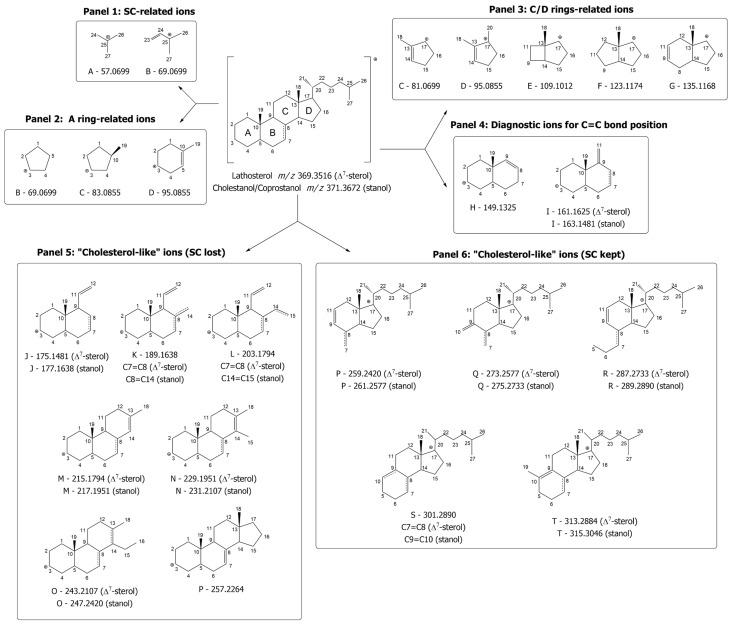
Proposed structures for product ions generated from the [M+H–H_2_O]^+^ ions of lathosterol, cholestanol, and coprostanol under HCD-MS/MS conditions. For the sake of simplicity, epimeric differences at C5 in stanols are not depicted. Exact *m*/*z* values are indicated for each ion. Unless otherwise noted, dashed-line double bonds are present only in lathosterol (∆^7^-sterol). See text for details.

While the HCD-MS/MS spectra of lathosterol and cholesterol (see Figure 3A in Ref. [49] for the latter) were broadly similar, some notable differences emerged. A prominent peak at *m*/*z* 69.0708 (consistent with an exact *m*/*z* 69.0699), attributed to a side chain-derived fragment (Panel 1, Scheme 1), was observed for lathosterol. This feature matched that observed for cluster B in Δ^7^-avenasterol (Figure 4E in Ref. [49]) and points to an alternative structure consistent with that *m*/*z* value, originating from ring A (Panel 2, Scheme 1), possible only if no C=C bond is located between C5 and C6. This makes the higher abundance of the *m*/*z* 69.0699 ion in cluster B a feature of Δ^7^-sterols and, as discussed later in this paper, also of sterols with no C=C bond on ring B. Actually, the presence of an unsaturation on the side chain, like in the case of desmosterol, can determine the formation of an alternative product ion with the same *m*/*z* ratio, thus increasing the corresponding peak signal (*vide infra*). Similarly to the *m*/*z* 69.0699 ion, the *m*/*z* 83.0861 one (exact *m*/*z* 83.0855), slightly prevailing in cluster C for both lathosterol (Figure 3A) and Δ^7^-avenasterol (Figure 4E, Ref. [49]), can also arise from ring A via cleavage of the C5–C10 and C4–C5 bonds, again requiring the absence of a C=C bond between C5 and C6. Notably, alternative side-chain-derived structures were proposed for the two ions for Δ^7^-avenasterol, yet their formation involved the presence of an ethylidene group at C24 (Scheme 3in Ref. [49]), a feature that is not present in lathosterol. Another shared feature between lathosterol and Δ^7^-avenasterol is the prominent *m*/*z* 95.0859 ion (exact *m*/*z* 95.0855) in cluster D. As evidenced in Panel 3 of Scheme 1, one of its possible structures is related to ring D (with the positive charge on C17) and is expected to be common to different sterols. However, an alternative structure involving ring A (Panel 2, Scheme 1) may also be formed in the case of Δ^7^-sterols via a retro-Diels–Alder reaction (see Figure S4), enabled by the presence of a C=C bond between C7 and C8. Clusters E–J displayed similar relative ion intensities for both cholesterol and lathosterol, with the exception of the *m*/*z* 149.1323 (exact *m*/*z* 149.1325) ion in cluster H, which was more abundant in Δ^7^-sterols, as confirmed in our prior study [49] and by Munger et al. [51]. Its most likely structure is reported in Panel 4 of Scheme 1. From clusters I to T, fragmentation profiles remained highly comparable between lathosterol and cholesterol, validating the previously proposed structures for the respective product ions [49]. The latter may arise either from neutral losses on the side chain, shared by both sterols, or from ring cleavages unaffected by the position of the double bond (see Panels 5 and 6, Scheme 1). The only significant exception in cluster similarity was the prevalence of the *m*/*z* 301.2880 ion (exact *m*/*z* 301.2890) in cluster S, in place of the *m*/*z* 299.2733 ion observed for cholesterol. As previously discussed [49], multiple structures can account for this ion. The one resulting from a breakage of the side chain, with the positive charge on C3 (see Scheme 1 in Ref. [49]), seems to be less relevant in the case of lathosterol. On the other hand, the alternative structure, with the positive charge on C17 and keeping the entire side chain (see Scheme 2 in Ref. [49]), is able to explain the detection of the *m*/*z* 301.2890 in the case of lathosterol considering only a C=C bond between C7 and C8. This is clarified by the structure reported in Panel 6 of Scheme 1 for this ion in the case of Δ^7^ sterols.

### 3.3. Fragmentation of Stanols with Saturated and Unbranched Side Chains: Coprostanol and Cholestanol

The APCI(+)-HCD-FTMS/MS spectra of the [M+H-H_2_O]^+^ ions of cholestanol and coprostanol (exact *m*/*z* 371.3672) are presented in Figure 3B and Figure 3C, respectively. As expected, the fragmentation profiles of these two epimeric sterols were virtually identical but the extent of fragmentation was more extensive than that of cholesterol, their mono-unsaturated analog, under equivalent collisional energy conditions. Clusters A to G included product ions with *m*/*z* values identical to those observed for cholesterol. This finding confirmed the structures previously proposed for them considering cholesterol [49], which are illustrated in Panels 1 and 3 of Scheme 1. Indeed, none of them include the C5=C6 double bond, which is the only structural difference between cholesterol and the two stanols. However, an important distinction was noted, since the ion at *m*/*z* 95.0859 (cluster D, exact *m*/*z* 95.0855) appeared with greater intensity in the case of stanols. As evidenced in Figure S5, the same structure reported for the *m*/*z* 95.0855 ion in the case of lathosterol (see Panel 2 in Scheme 1 and Figure S1), with the positive charge on C3, can be formed in the case of stanols through a retro-cycloaddition on ring B. This route complements the one previously proposed to account for that product ion in the case of cholesterol [49], with the positive charge located at C17 (see the structure reported in Panel 3, Scheme 1). The combined contributions of both pathways likely explain the enhanced signal intensity for the *m*/*z* 95.0855 ion in stanols. Additionally, as seen for lathosterol, alternative structures for ions at *m*/*z* 69.0699 and 83.0855 shown in Panel 2 of Scheme 1 are feasible in stanols due to the absence of a C5=C6 double bond, which would otherwise hinder such fragmentation.

The saturated nature of ring B in cholestanol and coprostanol was reflected in the *m*/*z* ratios found for ions in clusters H and I, compatible with exact values 149.1325 and 163.1481, respectively. Indeed, as evidenced in Panel 4 of Scheme 1, the structures proposed for those ions (consistent also with those reported by Munger et al. [51]) only included one C=C bond between C8 and C9, arising from the fragmentation process, but lacked the additional C=C bond at C5–C6 of cholesterol or at C7–C8 of Δ^7^-sterols. For clusters J to T, most stanol product ions demonstrated a consistent +2 shift in nominal *m*/*z* values compared to the corresponding cholesterol ions. The structures shown in Panels 5 and 6 of Scheme 1, which feature a positive charge at either C3 or C17, account for this shift as a result of saturation of the C5–C6 bond. This observation supports the structures previously proposed for cholesterol [49]. One notable exception to the +2 *m*/*z* shift was observed in cluster K, since the dominant product ion for stanols remained at a *m*/*z* value consistent with the exact value 189.1638, as for cholesterol. This indicates the formation of a second double bond along with the C11=C12 bond generated by ring C fragmentation. In lathosterol, this double bond is initially placed between C7 and C8, whereas in stanols it is proposed to be formed between C8 and C14 via a 1,3-hydrogen shift (see the structure in Panel 5 of Scheme 1). This hypothesis was confirmed by the detection of a weak peak signal at nominal *m*/*z* 187 in cluster K for lathosterol (see Figure 3A) and for cholesterol and Δ^5^/Δ^7^ phytosterols [49], since it would be consistent with the generation of the C8–C14 double bond, along with the one between C11 and C12, during fragmentation, and with the presence of the original double bond between C5–C6 or C7–C8. As expected, no such ion was detected for stanols (Figure 3B,C), which lack the necessary original C=C bond in ring B. A similar scenario was observed in cluster L, whose principal product ion was found at a *m*/*z* ratio consistent with the exact value 203.1794. As evidenced by the structure reported in Panel 5 of Scheme 1, this ion was likely generated through the formation of a C14=C15 double bond during fragmentation. This bond accounts for the −2 *m*/*z* units shift like the ring B double bonds characteristic of cholesterol and ∆^7^-sterols. The same process was likely responsible for the detection of a further product ion at nominal *m*/*z* 201 in cluster L both for lathosterol (see Figure 3A) and for cholesterol and Δ^5^/Δ^7^ phytosterols [49]. A third exception to the +2 *m*/*z* shift expected between lathosterol/cholesterol and stanols product ions was observed in cluster O, where the dominant stanol product ion appeared at *m*/*z* 247.2413 (exact *m*/*z* 247.2420), representing a +4 shift with respect to the lathosterol/cholesterol counterparts. As illustrated in Panel 5 of Scheme 1, this shift correlates with the absence of a C=C bond in ring B and reflects a modified fragmentation mechanism, ultimately keeping a single bond between C13 and C14.

It is finally worth noting that the distribution of relative intensities observed within most peak clusters detected in the HCD-FTMS/MS spectra of cholestanol and coprostanol [M+H–H_2_O]^+^ ions is coherent with that found for the respective clusters in MS/MS spectra acquired by Munger et al. [51] for the same sterols using electrospray ionization and a quadrupole-time of flight mass spectrometer. This finding, which was confirmed also for other sterols common between the two studies, suggests that the structure–fragmentation correlations inferred during the present investigation might be extended also to MS/MS spectra obtained under conditions different from those of HCD, provided they ensure an extensive fragmentation, including both the side chain and the steroidal core.

### 3.4. Fragmentation of Δ^5^- and Δ^8^-Sterols with a Double Bond at C24–C25: Desmosterol, Zymosterol and Lanosterol

**Scheme 2:**
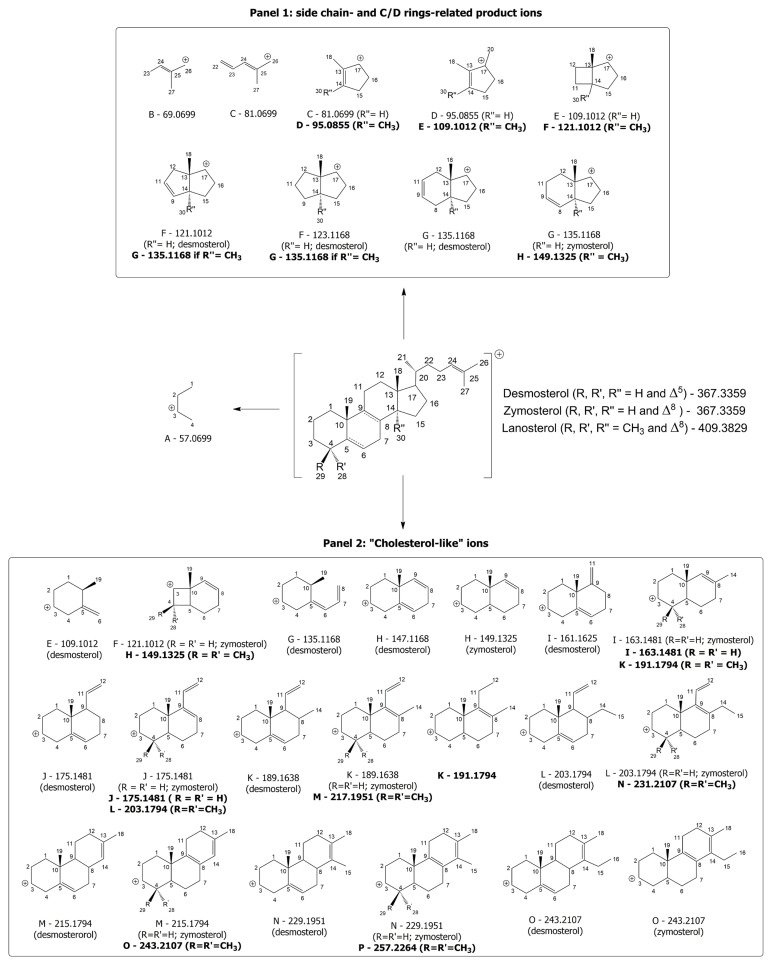
Proposed structures for major product ions detected in clusters A to O and A to P found in the APCI-HCD-MS/MS spectra of [M+H–H_2_O]^+^ ions of desmosterol/zymosterol and lanosterol, respectively. Exact *m*/*z* values are reported for all ions with four decimal places. The *m*/*z* values referring to common product ions for desmosterol and zymosterol are reported with plain character, without further specifications. The *m*/*z* values referring to product ions of lanosterol are reported with bold character, excepting those for product ions of clusters A, B and the non-cyclic product ion of cluster C, which are common to the three sterols. See text for details.

**Scheme 3:**
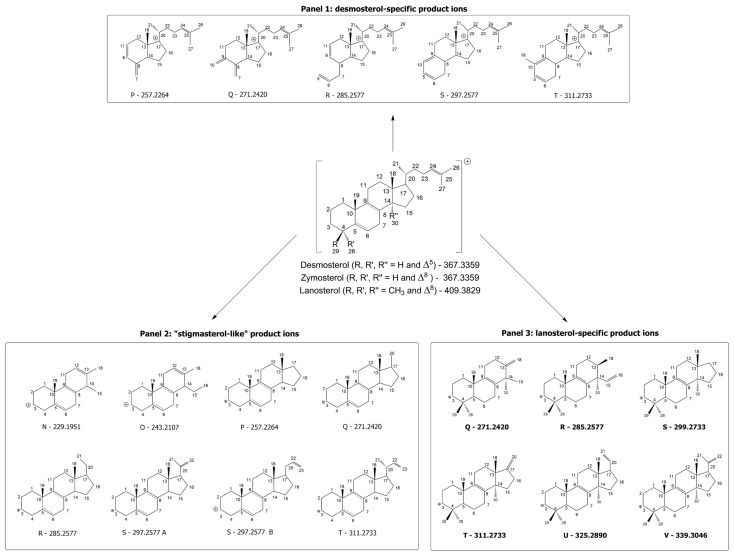
Proposed structures for major product ions detected in clusters N to T and Q to V found in the APCI-HCD-MS/MS spectra of [M+H–H_2_O]^+^ ions of desmosterol/zymosterol and lanosterol, respectively. Exact *m*/*z* values are reported for all ions with four decimal places. For structures reported in panel 2, which are common to desmosterol and zymosterol, the double bond inside ring B is meant to be present only in the respective correct position (C5–C6 and C8–C9, respectively). See text for details.

Desmosterol, zymosterol, and lanosterol were considered in this study as representative sterols featuring a C=C bond between C24 and C25. The APCI(+)-HCD-FTMS/MS spectrum of desmosterol (Figure 3D) was compared with that of cholesterol [49] to elucidate spectral features specific to Δ^5^-sterols carrying a C=C bond at C24–C25. Among low-*m*/*z* product ions, only one was detected in cluster A, at *m*/*z* 57.0708 (exact value: 57.0699). Due to the occurrence of the C=C bond at C24–C25, this ion cannot correspond to the t-butyl carbocation with the charge on C25 inferred by fragmentation of hexa-deuterated cholesterol [49]. Rather, its occurrence in desmosterol supports the alternative structure previously proposed for deuterated cholesterol [49], corresponding to an n-butyl carbocation with the charge on C3, detached from ring A (see Scheme 2). In cluster B, the dominant ion at *m*/*z* 69.0708 (compatible with an exact value 69.0699) reflects the presence of the C24–C25 unsaturation. This facilitates its generation as an allylic carbocation via cleavage of the C22–C23 bond, mediated by a 1,3-H transfer from C20 to C23 (Figure S3), with positive charge located on C26 or C27 (Scheme 2, Panel 1). Desmosterol product ions detected in clusters C to O align with structures previously assigned to cholesterol, as detailed in Scheme 2 (Panels 1 and 2). For clusters P to T, product ions were interpreted either by considering a cholesterol-related product ion with positive charge on C17, including the additional C=C bond at C24–C25 (Scheme 3, Panel 1), or by alternative structures, corresponding to those previously proposed for stigmasterol [49], featuring the positive charge on C3 and arising from partial side-chain loss (Scheme 3, Panel 2). Notably, like for cholestanol and coprostanol, the HCD-FTMS/MS spectrum of desmosterol was in excellent accordance, in terms of within-cluster intensity distribution, with the one obtained by Munger et al. using a Q-ToF mass spectrometer [51].

The APCI(+)-HCD-FTMS/MS spectrum of zymosterol ([M+H-H_2_O]^+^ precursor with exact *m*/*z* 367.3359; Figure 3E) was generally similar to that of desmosterol in terms of internal abundance profiles of clusters, with exceptions in clusters F, H, and I. In cluster F, the main ion became the one at *m*/*z* 121.1012, instead of desmosterol’s prevalent ion at *m*/*z* 123.1168. Mechanisms of formation proposed for the two ions in the case of cholesterol and phytosterols [49] could also be extended to desmosterol, leading to structures including rings C and D and with positive charge on C17 shown in Panel 1 of Scheme 2. However, those processes were hindered by the presence of a C=C bond between C8 and C9 in the case of zymosterol; thus a specific mechanism had to be hypothesized (see Figure S7) to explain the generation of the *m*/*z* 121.1012 ion from this sterol, with the proposed structure shown in Panel 2 of Scheme 2. As evidenced in Figure S7, the process likely involved the generation, as an intermediate, of an ion with exact *m*/*z* 149.1325, corresponding to the leading product ion detected at *m*/*z* 149.1323 in cluster H for zymosterol (see Figure 3E). A novel mechanism (see Figure S8) was also necessary to explain the generation of the ion detected at *m*/*z* 163.1479 (exact *m*/*z* 163.1481) in cluster I of zymosterol, leading to the structure reported in Panel 2 of Scheme 2. In fact, the mechanisms proposed in our previous paper and in Munger’s one [49,51] could not be applied to product ions prevailing in clusters H and I, since none of the sterols involved in the two studies was a Δ^8^ sterol, like zymosterol, and the presence of a C=C bond between C8 and C9 would have made those processes impossible.

Notably, despite an *m*/*z* 135.1167 ion dominating in cluster G for both zymosterol and desmosterol, a different structural interpretation was required for the two sterols. Structures previously proposed considering cholesterol (with positive charge either on C3 or on C17) [49] were consistent with the structure of desmosterol (see Panels 1 and 2 in Scheme 2) but incompatible with that of zymosterol due to the presence of the C8=C9 bond. Consequently, a new structure was proposed for the product ion in the case of zymosterol, including rings C and D and with the positive charge on C17 (see Panel 1 of Scheme 2).

Prevailing ions in clusters J to O for both sterols could be interpreted considering structures already proposed in the case of cholesterol [49] but, obviously, including the C=C bond on ring B between C5 and C6 for desmosterol and between C8 and C9 for zymosterol (Scheme 2, Panel 2). Finally, prevailing ions in clusters P to T for zymosterol could not be explained considering cholesterol-like structures, like those proposed for desmosterol (Scheme 3, Panel 1), due to the presence of the C8=C9 bond. On the other hand, stigmasterol-like structures (Scheme 3, Panel 2), previously proposed as alternatives for desmosterol ions of clusters N to T, could be extended also to zymosterol, simply considering the different location of the C=C bond in ring B.

Among Δ^8^-sterols considered in this study, lanosterol was chosen for its unique structural characteristics, related to the additional methylations on C4 (two methyl groups) and C14 (Figure 1). Not surprisingly, a distinct abundance profile was observed in its APCI(+)-HCD-MS/MS spectrum (Figure 4A). Despite this, lanosterol shared spectral features with zymosterol through cluster J, including identical *m*/*z* values for dominant ions. Prevailing ion in clusters A and B were assigned the same structures proposed for desmosterol and zymosterol (Scheme 2), as they arise from parts of molecular structure shared by the three sterols. Notably, the structure proposed for the *m*/*z* 69.0699 ion in Scheme 2 was the same as that hypothesised by Feng et al. [42] when studying the fragmentation of lanosterol, even though the transfer of positive charge from C3 to C26 (or C27), which is fundamental to generate that product ion, was not discussed in their paper.

As for cluster C, the prevailing ion could not be assigned the pentacyclic structure proposed before in this paper for desmosterol and zymosterol (Scheme 2, Panel 1, exact *m*/*z* 81.0699) due to the methylation at C14. Instead, the conjugated allylic carbocation with positive charge at C26 (Scheme 2, Panel 1), formed via a 1,3-H transfer from C23 to C20 and cleavage of the C20–C22 bond, could be easily assigned also to lanosterol. As apparent from Panel 1 of Scheme 2, prevailing product ions in clusters from D to H of lanosterol could be nicely explained considering structures proposed for ions in clusters from C to G for desmosterol/zymosterol (and, previously, for cholesterol and phytosterols [49]), all with the positive charge located on C17, just by taking into account the mass shift induced by the methylation of C14. This finding provides a strong further confirmation of the structures previously proposed for ions of clusters C to G in sterols non-methylated on C14. However, a further interpretation of the prevailing ion in cluster H for lanosterol (exact *m*/*z* 149.1325) could be found by considering the structure previously discussed for the major ion in cluster F of zymosterol (exact *m*/*z* 121.1012) and the double methylation on C4 (see Panel 2 of Scheme 2 and Figure S7). The potential existence of at least two routes for the generation of the *m*/*z* 149.1325 ion might be responsible for the remarkable intensity shown by its peak signal in the HCD-FTMS/MS spectrum of lanosterol (see Figure 4A), an almost unique feature in the MS/MS spectra of the sterols considered in this study.

A second striking feature of lanosterol’s spectrum was the prominent *m*/*z* 191.1797 ion in cluster K. Interestingly, Mo et al. [52] observed the same ion in the APCI(+)-CID-MS/MS spectrum of cycloartenol, which is perfectly isobaric with lanosterol, featuring two methyl groups on C4, missing the C=C bond between C8 and C9 and having a methylene bridge between C10 and C19 (corresponding to the methyl group at C19 in lanosterol). Based on the structural hypothesis proposed by Mo et al. [52], the prevailing ion in cluster K for lanosterol would correspond to the *m*/*z* 163.1481 ion of zymosterol with two methyl groups linked to C4, resulting in an exact *m*/*z* value 191.1794 (Scheme 2, Panel 2). However, the CID-MS^3^ analysis of this product ion of lanosterol, performed during this study using the Velos Pro linear ion trap mass spectrometer, revealed the generation of product ions identical to those found for the corresponding ion of cholesterol (nominal *m*/*z* 163, 149, 135, 121, 109, and 95), thus suggesting a possible alternative origin. A further structure was thus proposed (Scheme 2, Panel 2), involving an initial ethylene loss from the C4 dimethyl groups mediated by hydrogen migrations (Figure S9). This process could be fundamental to explain the formation of ions prevailing in clusters I and J of lanosterol (respective exact *m*/*z* 163.1481 and 175.1481), which would correspond to those reported for zymosterol in Panel 2 of Scheme 2. In fact, no other plausible route could be hypothesized for the generation of those product ions, considering the double methylation at C4. However, as evidenced in Scheme 2, plausible structures for product ions prevailing in clusters from L to P in the case of lanosterol could be obtained considering those proposed for clusters from J to N of zymosterol, respectively, and taking into account the presence of two methyl groups on C4. This finding emphasizes that the eventual preliminary loss of those methyl groups cannot be generalized to the entire population of lanosterol precursor ions. Finally, as emphasized in Panel 3 of Scheme 3, specific structures had to be hypothesized for product ions belonging to clusters from Q to V detected in the HCD-FTMS/MS spectrum of lanosterol, all of which exhibiting weak peak signals (see Figure 4A). They arose from fragmentations involving the side chain and then ring D, resembling those observed for cholesterol [49].

### 3.5. Fragmentation of Δ^5,7^-Sterols with a Double Bond at C22–C23: Ergosterol

**Scheme 4:**
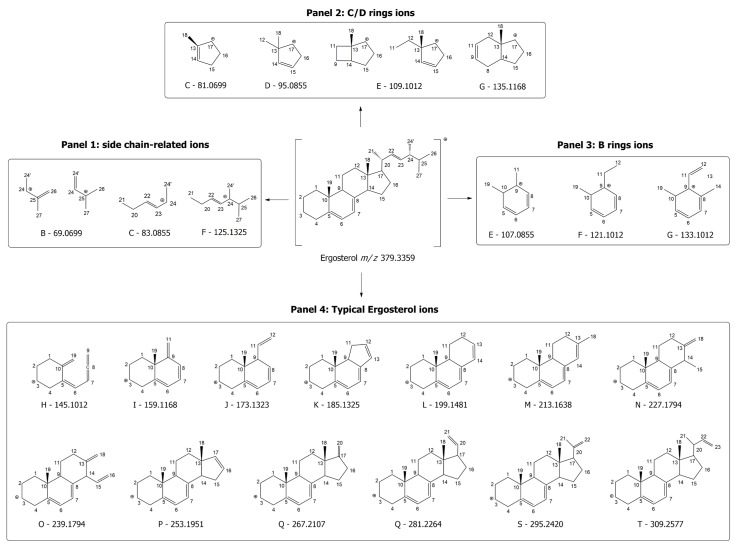
Proposed structures for product ions generated from the [M+H–H_2_O]^+^ ion of ergosterol (exact *m*/*z* 379.3359) under HCD-MS/MS conditions. Exact *m*/*z* values rounded to four decimal places are indicated for each ion. See text for details.

Ergosterol was selected in this study as a representative of Δ^5,7^-sterols. As shown in Figure 1, it features a double bond between C22 and C23 and a methyl group at C24, similarly to brassicasterol. The APCI(+)-HCD-FTMS/MS spectrum of the [M+H–H_2_O]*^+^* ion of ergosterol (exact *m*/*z* 379.3359), reported in Figure 4B, was dominated by the peak of cluster B at *m*/*z* 69.0708. This value is consistent with the exact value 69.0699, corresponding to a recurring product ion observed for sterols under HCD conditions (see also [49]), and thus also discussed in previous sections of this paper. As demonstrated in our previous study, considering stigmasterol pentadeuterated on its side chain and brassicasterol [49], this ion originates from the sterol side chain and is particularly prominent when a methyl (or ethyl) group is present at C24. In such configurations, C24 is both tertiary and allylic, facilitating positive charge stabilization. A plausible mechanism for its formation in the case of ergosterol involves a 1,3-hydrogen shift from C26 (or C27) to C24, followed by cleavage of the C23–C24 bond. This generates a stable allylic carbocation, shown on the left in Panel 1 of Scheme 4. Alternatively, if positive charge is located on the tertiary carbon atom C25, a 1,3-H shift from C24′ to C23 can yield another stable allylic/tertiary carbocation (Scheme 4, Panel 1). These processes can explain the high abundance of the *m*/*z* 69.0699 ion in ergosterol’s MS/MS spectrum.

The second most abundant product ion of ergosterol was detected in cluster C and its *m*/*z* value was consistent with the exact value 83.0855. Several structures could be proposed for this ion in the case of stigmasterol [49]; however, none of them is suitable to explain the ergosterol product ion, since this sterol has a methyl group, not an ethyl one, linked to C24. After evaluating multiple hypotheses, the structure depicted in Panel 1 of Scheme 4 emerged as the most likely. It assumes initial charge localization on C24 and involves two bond cleavages, between C24–C25 and C17–C20, facilitated by 1,3-H migrations (see Figure S10). This pathway results in a secondary/allylic carbocation. Furthermore, the dominant ion in cluster F (*m*/*z* 125.1325) likely serves as a precursor to the *m*/*z* 83.0855 ion, retaining the isopropyl moiety at the end of the side chain (Figure S7).

As shown in Figure 4B and corroborated by the intensity values reported in Table S1, all other product ions detected in the HCD-MS/MS spectrum of ergosterol fall below the 10% relative intensity threshold. This result suggests that the presence of conjugated double bonds in the B-ring reduces fragmentation along the steroidal backbone in favor of side-chain fragmentations, at least when a relatively high collisional energy is available, like under HCD conditions. Nevertheless, structures similar to those previously proposed for cholesterol [49], all bearing the positive charge on C17, could be hypothesised for product ions including rings C and/or D that were detected in clusters C, D, E and G, either as leading or as secondary ions (Panel 2, Scheme 3). On the other hand, novel structural hypotheses were required for the dominant product ions detected in clusters E (*m*/*z* 107.0858) and G (*m*/*z* 133.1010) for ergosterol. As illustrated in Panel 3 of Scheme 4, the presence of a double bond between C7 and C8 makes C9 an allylic and tertiary carbon atom, i.e., a viable site for positive charge localization.

A fragmentation involving partial cleavage of ring C and elimination of the left side of ring A as 1,3-butadiene may thus account for the formation of B-ring-related ions with exact *m*/*z* values 107.0855 and 133.1012 (see panel 3 of Scheme 4), consistent with experimental ones. Additionally, a secondary ion in cluster F (exact *m*/*z* 121.1012) may also arise from this mechanism (Scheme 4, Panel 3).

Product ions observed in clusters H, I, and P for ergosterol (consistent with exact *m*/*z* values 145.1012, 159.1168, and 253.1951, respectively) had been previously characterized by Munger et al. [51] as typical ergosterol product ions with positive charge retention at the original ionization site (C3). The same structures are thus depicted in Panel 4 of Scheme 4. Finally, it was possible to propose plausible structures for the dominant ions in all remaining clusters in ergosterol’s MS/MS spectrum, also displayed in Panel 4 of Scheme 3. These ions appear to result from progressive side-chain detachment and subsequent fragmentation of ring D, with the charge consistently residing at C3.

### 3.6. Fragmentation of Δ^5^-Sterols with a Double Bond at C24–C24′ (Methylene Group at C24): Chalinasterol

The final sterol examined in this study was chalinasterol, an isomer of brassicasterol. Chalinasterol is characterized by a methylene group at C24, i.e., a C=C double bond between C24 and C24′, whose influence on the fragmentation pattern was specifically investigated. As shown in Figure 4C, the APCI(+)-HCD-MS/MS spectrum of the [M+H–H_2_O]^+^ ion of chalinasterol (exact *m*/*z* 381.3516) reveals a dominant peak in cluster B, consistent with the recurring exact *m*/*z* value 69.0699. Figure S11 emphasizes that this ion can be readily formed if the positive charge is initially localized on C25, a carbon that is both tertiary and allylic in the case of chalinasterol. The formation process involves a typical 1,3-hydrogen migration, accompanied by cleavage of the C23–C24 bond.

For clusters C to F, the dominant ions correspond to structures previously proposed for cholesterol [49] but also reported for stanols and lathosterol in this paper (see Panel 3 of Scheme 1). These structures are appropriate here because they do not involve the side chain, where the unique structural feature of chalinasterol is located. Similarly, product ions dominating clusters G to O of chalinasterol can be explained using the same structures proposed for cholesterol and desmosterol (see Panel 2 of Scheme 2), since the side chain is not involved and the position of the C=C bond in ring B is identical.

A specific point of interest for chalinasterol is cluster P, where the ion at *m*/*z* 255.2107 slightly predominates. This represents a unique feature among the sterols analyzed in this study. In desmosterol, cluster P is dominated by an ion at *m*/*z* 257.2264, whose proposed structure is shown in Panel 3 of Scheme 2. However, due to differences in the side chain, this structure cannot explain the *m*/*z* 255.2107 ion observed for chalinasterol. One plausible structure for this ion could be obtained, in principle, considering the positive charge on C3 and a 1,3-hydrogen migration between C16 and C20, leading to cleavage of the C17–C20 bond and formation of a new C=C bond between C16 and C17. Mechanistically, this process should be feasible in all sterols considered so far. However, as evidenced in Figure 3 and Figure 4, and also in our previous work [49], this ion was not detected in other sterols, suggesting that the pathway described above is not generally favored. An alternative, more efficient route to generate the *m*/*z* 255.2107 ion when a C=C bond is located inside the side chain of sterols was suggested by Munger et al. [51]. These authors proposed that for sterols with a C=C bond at C24–C24′, such as chalinasterol (referred to in their work as 24-methylene-cholesterol), the *m*/*z* 255.2107 ion may arise from a retro-ene reaction. The presence of the C=C bond at C24–C24′ is essential for this reaction to proceed. Notably, this retro-ene process leads also to the formation of an intermediate product ion, which corresponds to the dominant peak observed in cluster S for chalinasterol (see Figure 4C). The structure of this ion is identical to that reported with the *m*/*z* label 297.2577 A in Panel 2 of Scheme 3, consistent with a stigmasterol-like product ion.

The structures proposed in the same panel for clusters Q to T, based on a B-ring double bond between C5 and C6, can be easily extended to chalinasterol. An additional noteworthy ion was detected for this sterol in cluster R, with a *m*/*z* value consistent with the exact one 283.2420. This product ion is expected to resemble the one assigned to cluster R in Panel 2 of Scheme 3 (*m*/*z* 285.2577), with the addition of a C=C bond between C20 and C21.

### 3.7. Identification of Sterols in Baker’s Yeast Based on Chromatographic and Tandem MS Data

**Figure 5:**
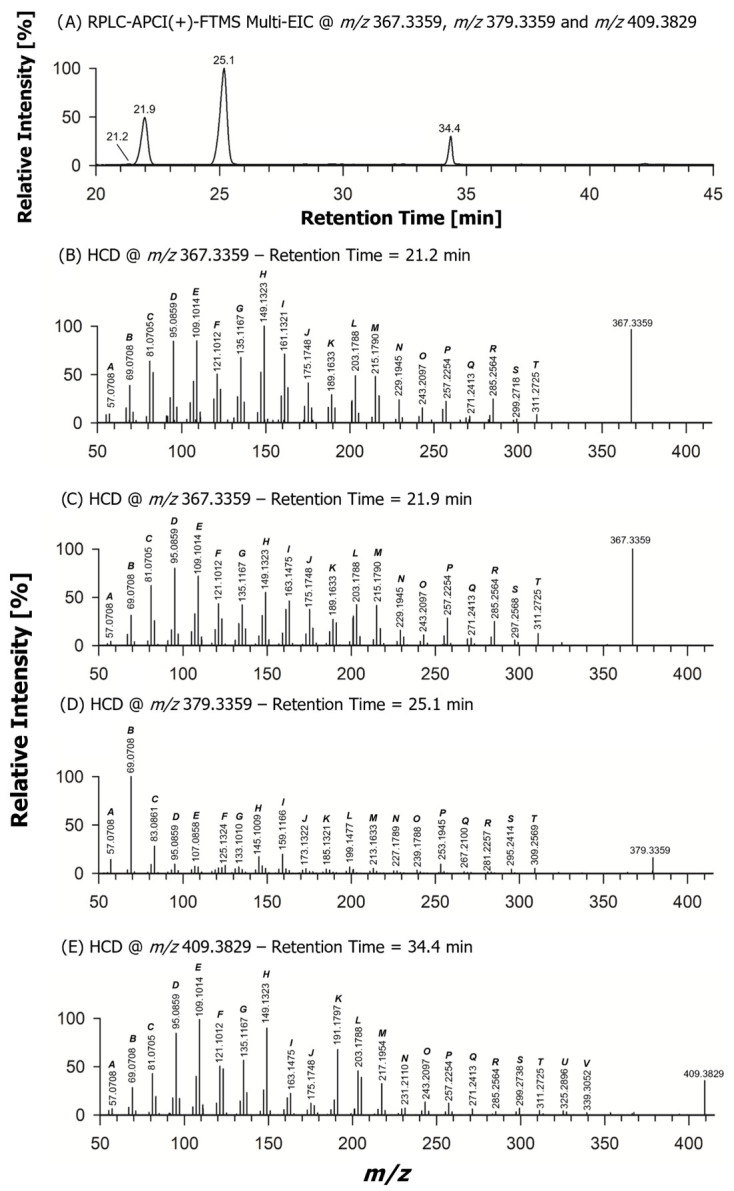
(**A**) Multiple-extracted ion current (EIC) chromatogram obtained from the RPLC-APCI(+)-FTMS analysis, performed under linear gradient conditions and using the Q Exactive mass spectrometer, of the sterol extract of baker’s yeast. (**B**–**E**) HCD-FTMS/MS spectra averaged under peaks detected, respectively, at 21.2, 21.9, 25.1 and 34.4 min in the chromatographic trace shown in panel A. The peaks were assigned, respectively, to 5-α-cholesta-7,24-dien-3-β-ol (the Δ^7^-analogue of zymosterol), zymosterol, ergosterol and lanosterol. See text for details.

To corroborate the robustness of HCD-MS/MS data obtained for standard sterol/stanols and thus verify the possibility of identifying these compounds in a real matrix through a combination of chromatographic and tandem mass spectrometric information, a sterol extract obtained from commercial baker’s yeast was subjected to RPLC-APCI(+)-FTMS and FTMS/MS analyses under the same conditions adopted in this work for standards. In particular, gradient elution was employed in the first step. FTMS spectra averaged under all peaks detected in the resulting Total Ion Current chromatogram were considered to evaluate the eventual presence of sterols/stanols based on the detection of the *m*/*z* values of their [M+H-H_2_O]^+^ ions. As a result, *m*/*z* ratios consistent with exact values 367.3359, 379.3359 and 409.3829 emerged and the corresponding multi-EIC trace was generated (see Figure 5A).

The comparison of retention times of peaks detected in the multi-EIC trace with those previously found, under linear gradient conditions, for animal/fungal standard sterols (see Figure 2B) indicated the occurrence of zymosterol (t_R_ = 21.9 min), ergosterol (t_R_ = 25.1 min) and lanosterol (t_R_ = 34.4 min). Actually, since standard zymosterol and desmosterol were found to be co-eluting under linear gradient conditions, an additional analysis of the baker’s yeast extract was performed under isocratic conditions, like those related to Figure 2A, ensuring separation between the two sterols. As a result, the presence of desmosterol in the extract was excluded. This outcome was also confirmed by the comparison of the APCI-FTMS/MS spectrum averaged under the peak eluting at 21.9 min, shown in Figure 5C, with the one obtained for standard zymosterol (Figure 3E). An excellent agreement was obtained, thus further excluding the occurrence of desmosterol, which, if present, would have led to an alteration of the spectral profile due to the interference caused by its product ions. A very good agreement was also found between APCI-FTMS/MS spectra averaged under peaks eluting at 25.1 and 34.4 min (Figure 5D and Figure 5E, respectively) and those obtained for standard ergosterol and lanosterol (Figure 4B and Figure 4A, respectively), thus confirming the identification based on retention time alignment.

The results discussed so far in this section highlight that the chromatographic and MS/MS information related to standard sterols/stanols can represent a solid base for their identification even in real complex matrices. Indeed, the possibility that other compounds co-extracted with sterols/stanols and almost isobaric with them are also co-eluted with them, thus leading to co-isolation and fragmentation of the respective ions, with a potential interference on the typical sterol/stanol MS/MS spectral profiles, is unlikely. Moreover, even if these unlikely circumstances occur, the MS/MS spectral profile of sterols/stanols is so characteristic, provided enough collisional energy is available, that at least a part of it would reasonably be unaffected by the interference due to a co-eluted, isobaric non sterolic/stanolic compound, thus still providing useful information.

Interestingly, the multi-EIC chromatogram obtained for the baker’s yeast extract evidenced the presence of a weak peak eluting just before the one related to zymosterol (t_R_ = 21.2 min) and not corresponding to any of the standards considered in this work and also in our previous work on cholesterol and phytosterols. The evaluation of the corresponding FTMS spectrum clearly showed that it was related to a sterol [M+H-H_2_O]^+^ ion with the same *m*/*z* value as zymosterol. The corresponding APCI-HCD-FTMS/MS spectrum, reported in Figure 5B, was then considered to infer information on its structural features. First, a good agreement with the intra-cluster profiles of the zymosterol MS/MS spectrum was obtained for clusters J through T, those related to the characteristics of the side chain, thus suggesting that the unknown compound included a side chain with a C=C bond between C24 and C25. As for the steroidal backbone, key information was inferred from clusters C, D and H, in which product ions consistent with exact *m*/*z* 83.0855, 95.0855 and 149.1325 exhibited relevant intensities. As discussed before, and as further evidenced in the next section of this paper and in Table 1, these spectral features are all coherent with the occurrence of a C=C bond between C7 and C8. Based on these considerations, the unknown sterol was tentatively assigned as the Δ^7^ analogue of zymosterol, nominally 5-alpha-cholesta-7,24-dien-3-β-ol, which is known to be a biosynthetic precursor of ergosterol [53]. As discussed before, the latter was clearly detected in the baker’s yeast extract, being a typical fungal sterol. Notably, chromatographic information was consistent with the tentative identification of the unknown sterol as the Δ^7^ analogue of zymosterol. Indeed, it was eluted slightly before zymosterol under linear gradient conditions, which also means that it would have been eluted slightly before desmosterol, its Δ^5^ analogue, if this sterol had been present, with zymosterol and desmosterol co-eluting under those conditions. In other words, the presumed Δ^7^ analogue of zymosterol/desmosterol exhibited a slightly lower retention time compared to its Δ^5^ counterpart. This finding is in accordance with the relative retention under linear gradient conditions of lathosterol and cholesterol (Δ^7^ vs Δ^5^), evidenced during this work, and of Δ^7^ and Δ^5^ avenasterols, emerged from our previous study [49]. The procedure described for the identification of the ergosterol precursor in the baker’s yeast extract emphasizes how important the evaluation of MS/MS data on sterols/stanols can be, especially if integrated with chromatographic information, when compounds not corresponding to major sterols/stanols, whose standards might be unavailable, emerge from the analysis of real complex matrices.

### 3.8. Correlation Between Specific Product Ions and Structural Features of Major Animal, Fungal and Vegetal Sterols

As highlighted in Figure 1, the principal animal and fungal sterols differ structurally in several respects. These include variations in the composition and branching of the side chain, the number and positional isomerism of C=C double bonds along the steroidal backbone, and the presence of additional methyl groups, such as those at C4 and C14 in the case of lanosterol. The systematic evaluation of their MS/MS spectral profiles, as presented in the preceding sections, revealed clear correlations between these molecular features and the fragmentation pathways. These correlations are consistent with patterns previously identified for cholesterol and the major plant sterols [49], where relative intensities of diagnostic product ions were shown to reflect specific structural characteristics.

To consolidate these findings, a summary of the structure–product ion correlations inferred after carefully evaluating the HCD-MS/MS spectra obtained both for animal/fungal sterol considered in the present study and for cholesterol and phytosterols considered in Ref. [49] is reported in Table 1. The correlations are evidenced following the increasing order of product ions *m*/*z* values, thus spanning clusters B through T; cluster A is not included, as its ions are not structurally informative. It is worth noting that the indications reported in Table 1 are those that were found to be consistent between animal, fungal and plant sterols/stanols when considering compounds with the same structural characteristics. In the last four columns, the table also reports an indication of the most likely origin of a specific product ion, i.e., if it is expected to be formed at the level of the side chain or the steroidal backbone (in the case of lighter ions) and, from cluster H on, if its structure can be assimilated to the one of a product ion with the same *m*/*z* generated from cholesterol or stigmasterol, as inferred during our previous study, also including isotopically labelled analogues of these two sterols [49]. Table 1 might be employed as a practical tool for interpreting the MS/MS spectra of unknown sterols that do not match with available standards but may be present in various animal, fungal or vegetal matrices. In particular, the relative intensities of ions within each cluster can offer valuable information about the number and position of unsaturations, the structure of the side chain, and the occurrence of specific methylations or branching patterns.

**Figure 6:**
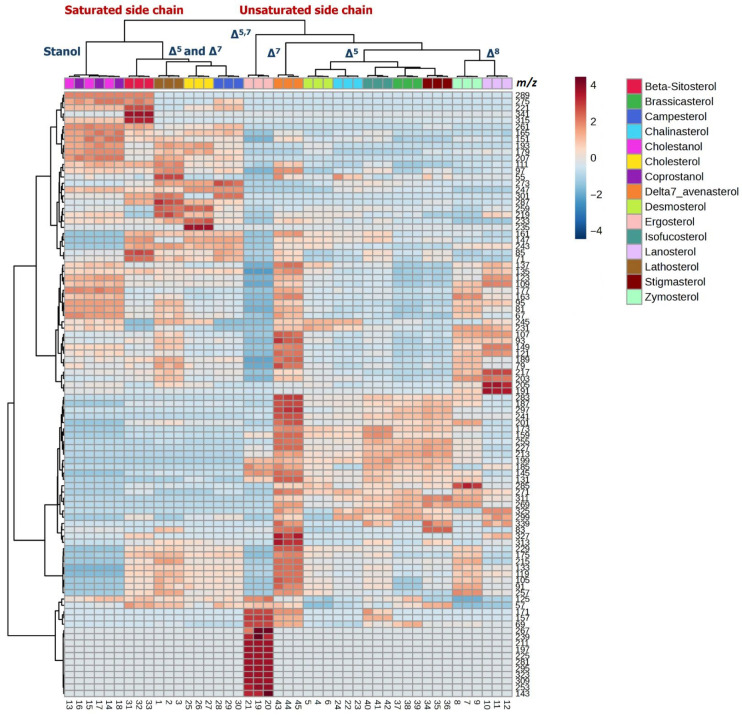
Double dendrogram (horizontal for objects and vertical for variables) with heatmap obtained from the Hierarchical Cluster Analysis of relative intensity data referring to product ions detected in APCI(+)-HCD-FTMS/MS spectra of animal/fungal sterols/stanols analyzed in the present study and cholesterol and phytosterols considered in Ref. [49]. Each compound was analyzed in triplicate; thus, three small squares refer to each compound at the base of the object dendrogram. Major structural characteristics related to groups or single compounds, namely the saturation or unsaturation of the side chain and the absence (stanols) or presence of C=C bonds (Δ) on the steroidal backbone and on the side chain, are evidenced in the object dendrogram. Numbers reported at the end of variable rows represent the nominal *m*/*z* ratios of the corresponding product ions that were used as variables for HCA.

As an additional step to emphasize the consistency of structure–fragmentation correlations reported in Table 1 for sterols/stanols of different origins, MS/MS data obtained for compounds considered in this study and in Ref. [49] were subjected to processing based on well-known chemometrical unsupervised approaches, namely Hierarchical Cluster Analysis (HCA) and Principal Components Analysis (PCA). With this aim, besides the sterols/stanols considered in this study, standard cholesterol and phytosterols considered in Ref. [49] were subjected to DIA-APCI(+)-HCD-FTMS/MS analysis in triplicate, under the same experimental conditions. The relative intensities of product ions inferred from each spectrum were then stored in a dataset and HCA and PCA were performed considering each compound as a statistical object and each product ion as a variable, labelled with the corresponding nominal *m*/*z* ratio. The relative intensity obtained for each product ion in each spectrum was considered as its value as an input variable for HCA and PCA. The values were preliminarily autoscaled and then HCA and PCA were performed using the Metaboanalyst 6.0 software, freely accessible on the Internet (https://www.metaboanalyst.ca/). In the case of HCA, Euclidean distances and the Ward agglomeration algorithm were employed for clustering. The outcome of HCA, consisting of a double dendrogram (horizontal for objects and vertical for variables) and the corresponding heatmap, is reported in Figure 6. The outcome of PCA, represented by the scores and loadings plots, is reported in Figure S12 of the Supplementary Materials.

First, the already discussed reproducibility of MS/MS spectral relative intensities can be inferred by the proximity of symbols corresponding to the three replicates for each compound in the PCA scores plot and by the generally excellent clustering of replicates for a specific sterol/stanol observed in the HCA dendrogram, with the only exception occurring for the two stanols due to the remarkable similarity of their MS/MS spectra. Moreover, as emphasized by labels added to the horizontal dendrogram in Figure 6, HCA on MS/MS relative intensity data was able to create clusters and sub-clusters clearly related to structural features. Indeed, sterols/stanols were clearly separated in the two major clusters, based on the nature of the side chain, i.e., saturated vs. unsaturated. The cluster of compounds with a saturated side chain was then sub-clustered according to the saturation (stanols) or unsaturation (Δ^5^ and Δ^7^ sterols) of the steroidal backbone. On the other hand, the major cluster of sterols with an unsaturated side chain was nicely sub-clustered according to the position and the number of C=C bonds on the steroidal backbone. Indeed, ergosterol represented a single sub-cluster, due to its unique double unsaturation on the backbone among the studied compounds, determining a peculiar profile of product ions, clearly visible in the HCA heatmap. Δ^7^-avenasterol was also separated as a single cluster, whereas the two Δ^8^ sterols (zymosterol and lanosterol) were surprisingly grouped together despite the additional triple methylation characterizing lanosterol. This outcome suggests that the identical position of the C=C bond on the steroidal backbone and the identity of the side chains was more important in determining the fragmentation pathways of the two sterols than those methylations. Finally, all Δ^5^ sterols with unsaturated side chain were sub-clustered together in the dendrogram (see Figure 6) and then their sub-cluster was further split in two groups, reflecting the position of the C=C bond on the side chain, namely, the proximity of that bond to the steroidal backbone, thus indicating how refined can be the influence of structural features on the profile of MS/MS spectra.

The interesting information arising from HCA was confirmed by PCA. Indeed, as emphasized in Figure S12, all compounds including a saturated side chain were grouped in the lower left quadrant of the score plot, with scores referring to stanols being very close, as expected, although symbols corresponding to the respective replicated samples were not mixed like in the case of HCA. On the other hand, sterols with C=C bonds on the side chain were located at positive values of PC2 and then, once again, separated according to the position of the unsaturation on the steroidal backbone. Finally, as observed in the HCA dendrogram, ergosterol and Δ^7^-avenasterol were located in characteristic positions of the plot due to their very specific structural features.

The outcomes of HCA and PCA on HCD-MS/MS data strengthen the concept that an extensive fragmentation of sterol/stanol [M+H-H_2_O]^+^ ions, achievable by a careful tuning of the collisional energy, according to the available tandem MS instrumentation, can provide a remarkable amount of information and open interesting perspectives for the recognition of the structural features of novel or poorly characterized sterols in complex matrices.

## 4. Conclusions

Tandem mass spectrometry under HCD conditions, applied to [M+H–H_2_O]^+^ ions generated via APCI, proved highly effective for characterizing representative animal and fungal sterols, as previously shown for cholesterol and major plant sterols. Despite the inherent spectral complexity, marked by the detection of recurring fragment clusters, a systematic interpretation was enabled by considering alternative locations of the positive charge with respect to the original position (C3), in particular allylic or tertiary carbocations. This allowed for the proposal of plausible structures for major product ions and revealed consistent correlations between their relative abundances and specific structural features, including the position of C=C bonds, side chain alkylation, and additional methylations (like those occurring in lanosterol). These fragmentation trends closely mirrored those previously observed for cholesterol and plant sterols. This consistency was confirmed by the chemometric processing, through HCA and PCA, of MS/MS spectral data obtained for the entire ensemble of compounds, including them and the animal/fungal sterol/stanols considered in this study. Indeed, chemometrics outcomes emphasized the similarity in fragmentation behavior in the presence of common structural features, regardless of the origin of the compounds under study. Finally, the application of RPLC-APCI(+)-HCD-FTMS/MS analysis to the sterol extract of baker’s yeast, selected as an example of a complex matrix, confirmed the power of MS/MS data, along with chromatographic outcomes, in terms of recognition of novel sterols isomeric with known ones, with a Δ^7^ analogue of zymosterol being identified along with the latter and other well-known fungal sterols.

Overall, this study offers a comprehensive framework for interpreting MS/MS spectra of diverse free sterols/stanols, paving the way for the structural elucidation of novel compounds of those classes in complex matrices.

## Figures and Tables

**Table 1:** Summary of the possible correlations between structural features of sterols and stanols, namely C=C bond(s) position(s) and side chain type, and intensity relationships between product ions belonging to specific signal clusters detected in the HCD-FTMS/MS spectra (acquired at NCE = 30) of [M+H-H_2_O]^+^ ions of standard animal/vegetal/fungal sterols and stanols discussed in the present paper and in Ref. [49]. The tentative assignment of a product ion to a specific portion of the sterol/stanol molecule or the similarity with the corresponding product ion previously detected in the case of cholesterol or stigmasterol^49^ are evidenced in the last four columns. Exact *m*/*z* values, rounded to four decimal places, are reported for each ion. The name “stanol” in the table is referred to stanols with the same side chain as cholesterol.

Cluster	Exact *m*/*z* Values	Intensity Relationships	Type of Product Ion and Related Structural Features			
			Side Chain- Related Ion	A/B or C/D Rings Ion	Cholesterol- Like Ion	Stigmasterol- Like Ion
B	69.0699	69.0699 >> 71.0855	Δ^24(24′)^, Δ^24(25)^ Δ^22(23)^, Δ^5,22(23)^	Δ^7^ and stanols		
	71.0855	71.0855 > 69.0699	Δ^5^ with saturated SC			
C	81.0699	81.0699 > 83.0855		Δ^5^ with saturated SC Δ^24(24′)^, Δ^24(25)^, and stanols		
	83.0855	83.0855 > 81.0699	Δ^22(23)^ alkylated at C24			
		83.0855 > 50%		Δ^7^		
D	95.0855	95.0855 > 80%		Δ^7^ and stanols		
F	121.1012	121.1012 > 123.1168		Δ^5,22(23)^, Δ^5^ and Δ^7^ with ethylene group at C24, and Δ^8,24(25)^		
	123.1168	123.1168 > 121.1012		Δ^5^ and Δ^7^ with saturated SC, stanols, Δ^5,24(25)^, Δ^5^ with methylene group at C24		
	125.1325	125.1325 > 123.1168	Δ^5,7,22(23)^ methylated at C24			
G	133.1012	133.1012 > 135.1168		Δ^5,7^		
H	145.1012	145.1012 > 147.1168			Δ^5,7^	
	147.1168	147.1168 >> 149.1325			Δ^5^	
	149.1325	149.1325 >> 147.1168			Δ^7^, Δ^8^ and stanols	
I	159.1168	159.1168 > 161.1325			Δ^5,7^	
	161.1325	161.1325 >> 163.1481			Δ^5^ and Δ^7^	
	163.1481	163.1481 > 161.1325			Δ^8^ and stanols	
J	173.1324	173.1324 > 175.1481				Δ^22(23)^
	175.1481	175.1481 >> 173.1324			Δ^24(24′)^, Δ^24(25)^ and sterols with saturated SC	
	177.1638	177.1638 > 175.1481			stanols	
K	185.1325	185.1325 > 187.1481				Δ^5,7,22(23)^
	187.1481	187.1481 > 189.1638				Δ^22(23)^
	189.1638	189.1638 > 187.1481			Δ^24(25)^, sterols with saturated SC and stanols	
	191.1794	191.1794 >> 189.1638			4,4-dimethyl-Δ^8,24(25)^	
L	199.1481	199.1481 > 201.1638				Δ^5,7,22(23)^
	201.1638	201.1638 > 203.1794				Δ^22(23)^
	203.1794	203.1794 ≥ 205.1951			4,4-dimethyl-Δ^8,24(25)^	
M	213.1638	213.1638 > 215.1790			Δ^5,7^	
	217.1951	217.1951 > 215.1790			4,4-dimethyl-Δ^8^ and stanols	
N	227.1794	227.1794 > 229.1946				Δ^22(23)^
	229.1946	229.1946 > 227.1794			Δ^5^ and Δ^7^ with saturated SC, Δ^24(24′)^ and Δ^24(25)^	
	231.2107	231.2107 > 229.1946			4,4-dimethyl-Δ^8,24(25)^ and stanols	
O	239.1794	239.1794 > 241.1951				Δ^5,7,22(23)^
	241.1951	241.1951 > 243.2107				Δ^22(23)^ and Δ^24(24′)^ with ethylene group at C24
	243.2107	243.2107 > 241.1951			sterols with saturated SC, stanols, Δ^24(24′)^ with methylene group at C24 and Δ^24(25)^	
	247.2420	247.2420 > 243.2107			stanols	
P	253.1951	253.1951 > 255.2103				Δ^5,7,22(23)^
	255.2103	255.2103 ≥ 257.2269				Δ^22(23)^ and Δ^24(24′)^ with ethylene group at C24
	257.2269	257.2269 > 259.2426			sterols with saturated SC alkylated at C24, Δ^7^ with saturated unbranched SC	Δ^24(25)^
	259.2426	259.2426 > 257.2269			Δ^5^ with saturated unbranched SC	
	261.2577	261.2577 > 259.2426			stanols	
R	281.2264	281.2264 > 283.2420				Δ^5,7,22(23)^
	283.2420	283.2420 > 285.2577				Δ^22(23)^ and Δ^24(24′)^
	285.2577	285.2577 > 283.2420				Δ^24(25)^
	287.2733	287.2733 > 283.2420			sterols with saturated SC	
	289.2890	289.2890 > 287.2733			stanols	
S	295.2420	295.2420 > 297.2577				Δ^5,7,22(23)^
	297.2577	297.2577 > 299.2739				Δ^24(24′)^ and Δ^24(25)^
	299.2733	299.2739 > 297.2577			Δ^5^-with saturated and unbranched SC. 4,4-dimethyl-Δ^8,24(25)^	
	301.2890	301.2890 > 299.2733			Δ^7^-and stanols	
T	309.2577	309.2577 > 311.2733				Δ^5,7,22(23)^
	311.2733	311.2733 > 313.2890				Δ^5,22(23)^, Δ^5,24(24′)^ and Δ^24(25)^
	315.3046	315.3046 > 313.2890			Δ^5^ with ethyl group at C24, stanols	

## Data Availability

The data presented in this study are available on request from the corresponding author.

## Supplementary Materials

The following supporting information can be downloaded at https://www.mdpi.com/article/10.3390/metabo15100674/s1, Figures S1–S3: APCI(+)-HCD-FTMS/MS spectra obtained using the Q Exactive mass spectrometer for the [M+H–H_2_O]^+^ ions of sterols and stanols considering NCE values 10/50/70; Figure S4: proposed mechanism for the generation of the *m*/*z* 95.0855 ion from Δ^7^-sterols; Figure S5: proposed mechanism for the generation of the *m*/*z* 95.0855 ion from stanols; Figure S6: proposed mechanism for the generation of the *m*/*z* 69.0699 ion from desmosterol, zymosterol and lanosterol; Figure S7: proposed mechanism for the generation of ions with exact *m*/*z* 149.1325/177.1638 and 121.1012/149.1325 from zymosterol/lanosterol; Figure S8: proposed mechanism for the generation of the *m*/*z* 163.1481 ion from zymosterol; Figure S9: proposed mechanism for the gas-phase demethylation at C4 of lanosterol; Figure S10: proposed mechanism for the generation of ions with exact *m*/*z* 83.0855 and 125.1325 from ergosterol; Figure S11: proposed mechanism for the generation of the *m*/*z* 69.0699 ion from chalinasterol; Figure S12: scores and loading plots obtained upon principal components analysis based on the HCD-MS/MS spectral relative intensities for animal, fungal and vegetal sterols and stanols. Table S1: summary of chromatographic resolution and selectivity values evaluated for couples of close sterol/stanol peaks after the RPLC-APCI(+)-FTMS analysis of their standard mixture performed under isocratic or linear gradient conditions; Table S2: summary of mass spectrometric information, i.e., experimental *m*/*z* values, assigned molecular formulas with related mass accuracy and mean values (±standard deviations) of relative intensities (*n* = 3) concerning product ions detected in the APCI(+)-HCD-FTMS/MS spectra of the [M+H−H_2_O]^+^ ions of sterols/stanols analyzed in this study.

## Abbreviations

The following abbreviations are used in this manuscript:

ACN	ACetoNitrile
AGC	Automatic Gain Control
APCI	Atmospheric Pressure Chemical Ionization
CID	Collisionally Induced Dissociation
DIA	Direct Infusion Analysis
EIC	Extracted Ion Current
ESI	ElectroSpray Ionization
EtOH	Ethanol
FA	Formic Acid
FTMS	Fourier-Transform Mass Spectrometry
GC-EI-MS	Gas Chromatography—Electron Ionization—Mass spectrometry
HCD	Higher-Collisional-energy Dissociation
HPLC	High-Performance Liquid Chromatography
HRMS	High-Resolution Mass Spectrometry
HRMS/MS	High-Resolution Tandem Mass Spectrometry
IUPAC	International Union of Pure and Applied Chemistry
MS^n^	Multistage Mass Spectrometry
NCE	Normalized Collisional Energy
RF	Radio Frequency
RPLC	Reversed-Phase Liquid Chromatography
SC	Side Chain
SMT	Sterol Methyl-Trasferase
