# Supplementary material for: Diagnostic Fragmentations of Animal and Fungal Sterols/Stanols Obtained by APCI–Tandem Mass Spectrometry: A Route Towards Unknown Free Sterol Identification
Source: Metabolites. 2025 Oct 16;15(10):674. doi: 10.3390/metabo15100674 (PMC12565904; doi:10.3390/metabo15100674)
Supplement: Supplementary file 1 [file metabolites-15-00674-s001.zip › metabolites-3855604-supplementary.pdf]

Supplementary Materials

Diagnostic fragmentations of animal and fungal sterols/stanols obtained by APCI-Tandem Mass Spectrometry: a route towards unknown free sterols identification

V. Cinquepalmi[a], I. Losito[a,b,*], A. Castellaneta[a], C.D. Calvano[a,b], T.R.I. Cataldi[a,b]

[a]*Dipartimento di Chimica and* [b]*Centro Interdipartimentale SMART – Università degli Studi di Bari, via E. Orabona 4, Bari;*

Number of Figures: 12

Number of Tables: 2

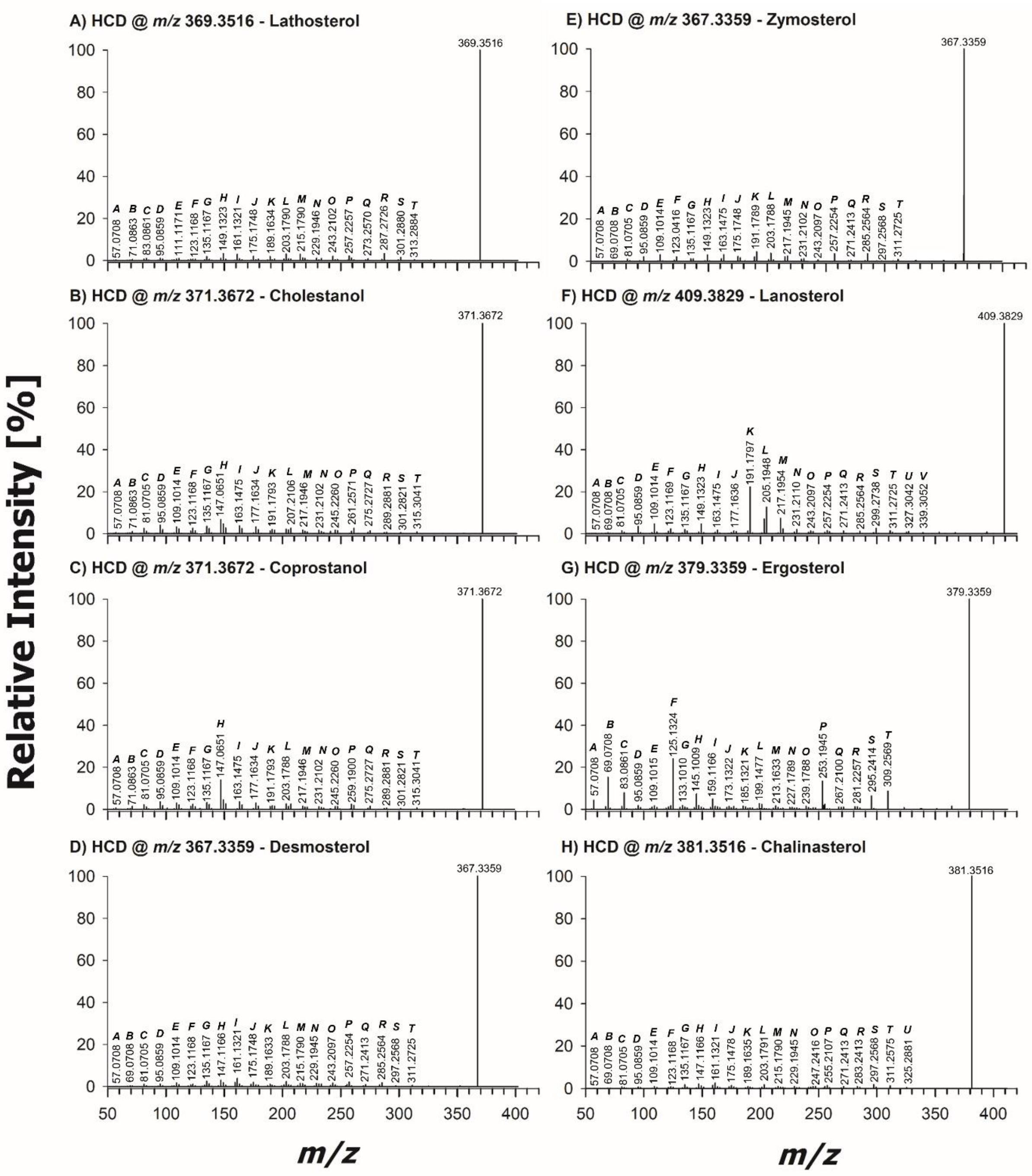

Figure S1. APCI(+)-HCD-FTMS/MS spectra obtained using the Q Exactive mass spectrometer for the $[M+H–H_2O]^+$ ions of sterols and stanols analyzed in this study considering a Normalized Collisional Energy (NCE) value equal to 10. Peak signals referred to product ions sharing the same number of carbon atoms but differing for the number of H atoms were grouped into clusters, each labelled with a capital letter. For the sake of clarity, the experimental *m/z* value of only one ion (usually the most abundant) is reported for each cluster.

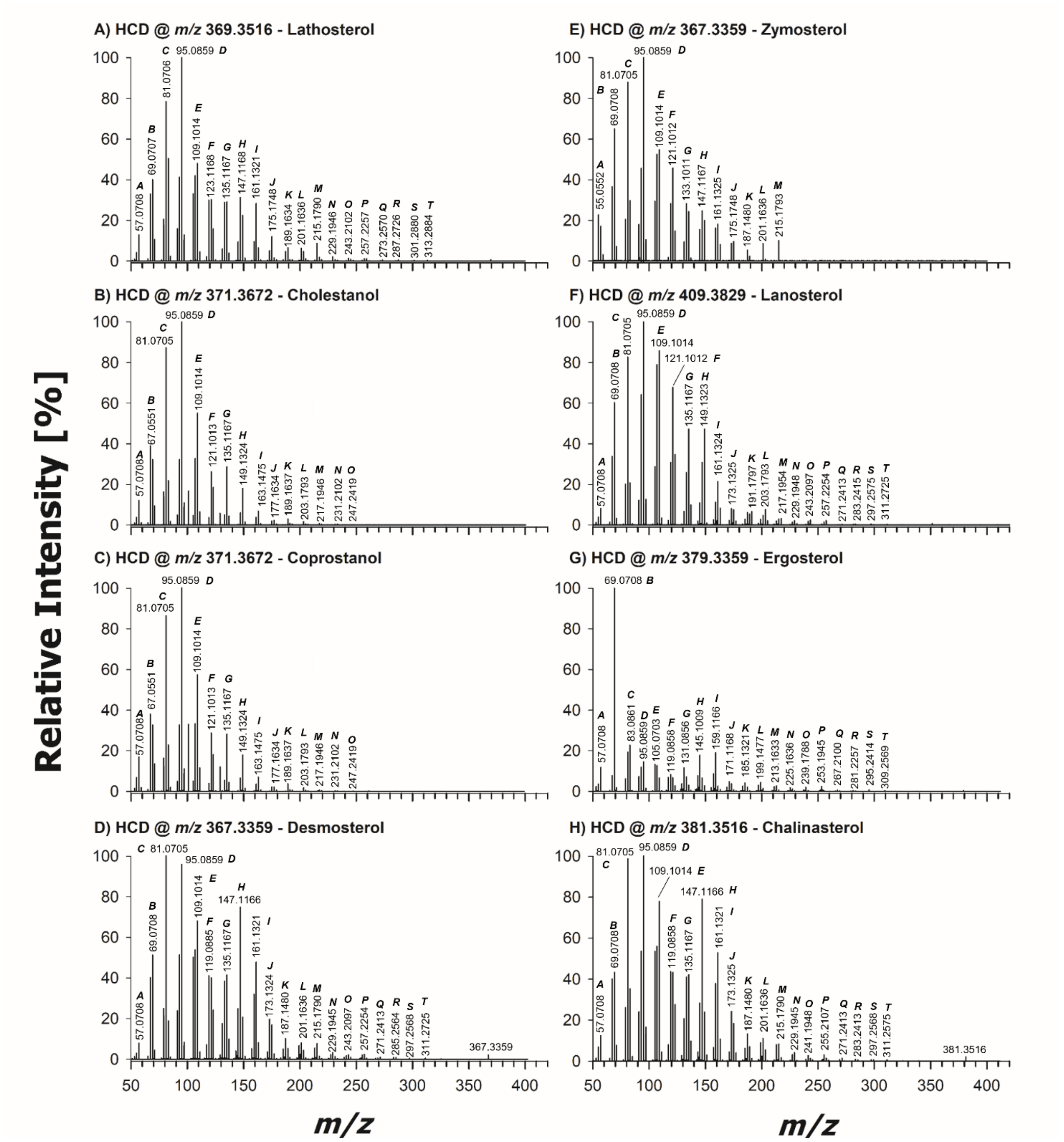

Figure S2. APCI(+)-HCD-FTMS/MS spectra obtained using the Q Exactive mass spectrometer for the $[M+H-H_2O]^+$ ions of sterols and stanols analyzed in this study considering a NCE value equal to 50. Peak signals referred to product ions sharing the same number of carbon atoms but differing for the number of H atoms were grouped into clusters, each labelled with a capital letter. For the sake of clarity, the experimental *m/z* value of only one ion (usually the most abundant) is reported for each cluster.

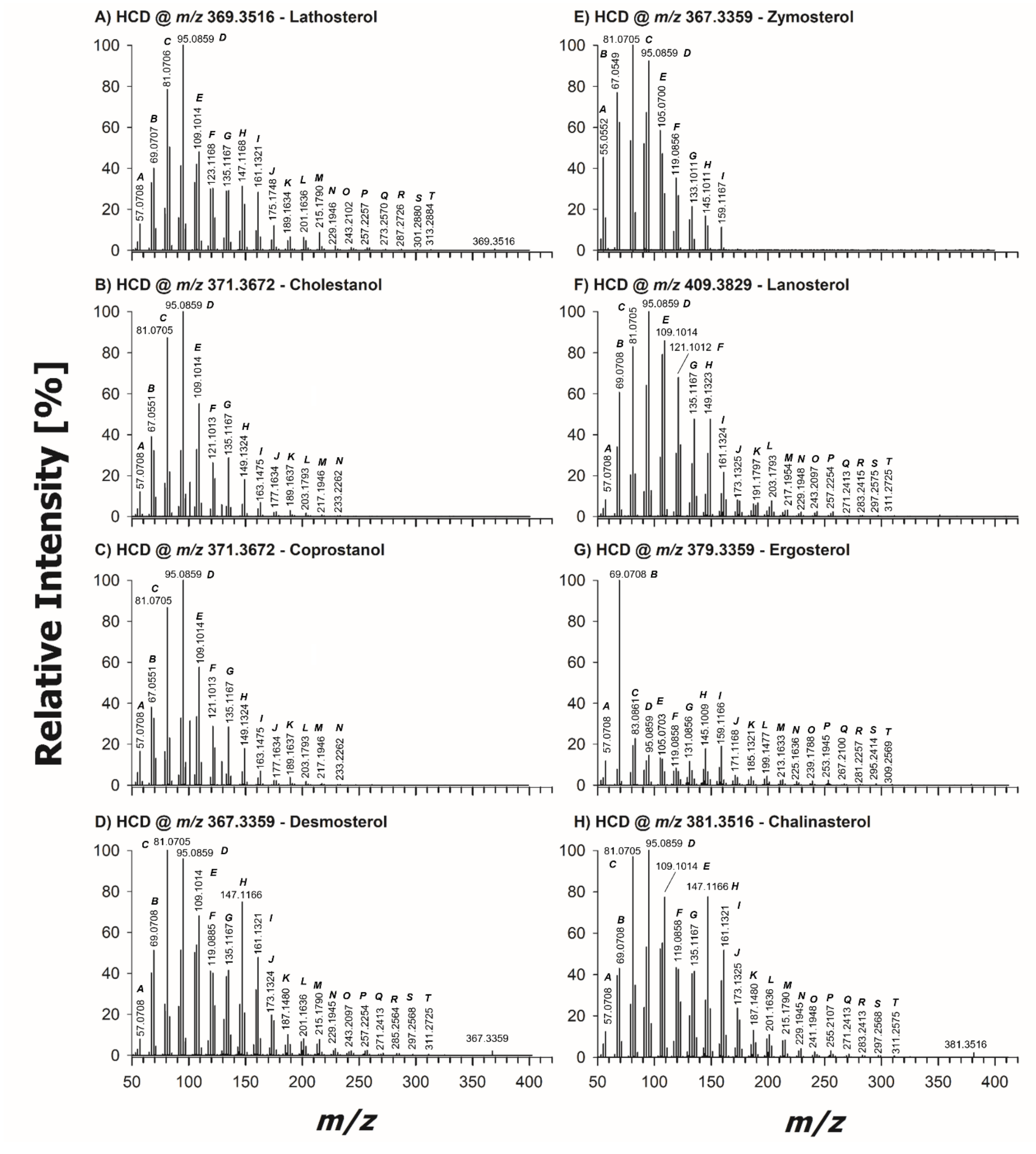

Figure S3. APCI(+)-HCD-FTMS/MS spectra obtained using the Q Exactive mass spectrometer for the $[M+H–H_2O]^+$ ions of sterols and stanols analyzed in this study considering a NCE value equal to 70. Peak signals referred to product ions sharing the same number of carbon atoms but differing for the number of H atoms were grouped into clusters, each labelled with a capital letter. For the sake of clarity, the experimental *m/z* value of only one ion (usually the most abundant) is reported for each cluster.

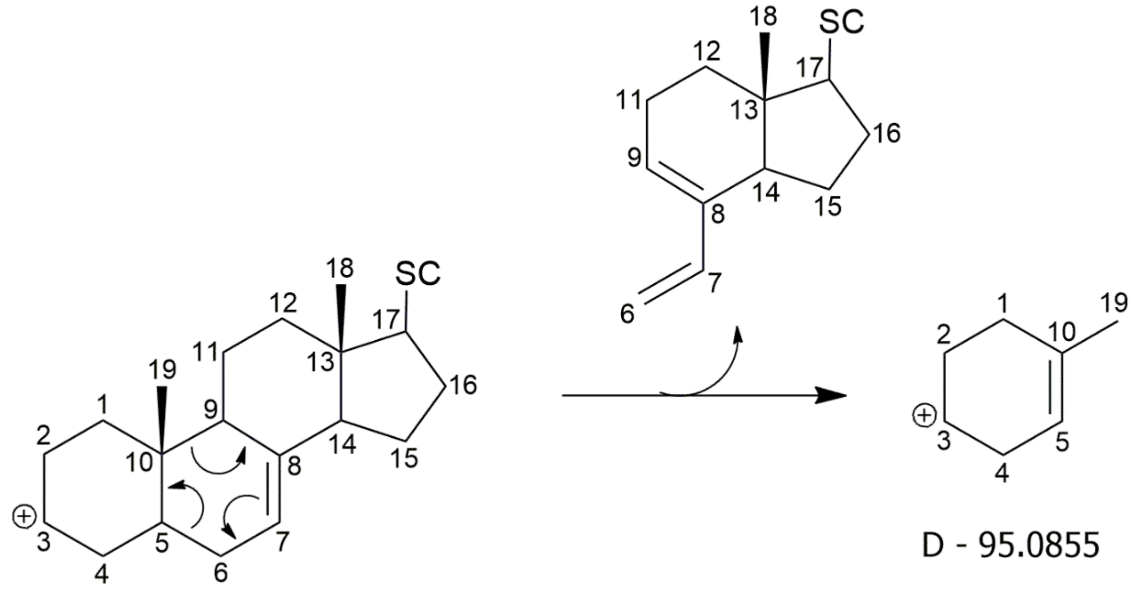

Figure S4. Proposed mechanism for the gas-phase formation of an A-ring-derived product ion, detected in cluster D, from the $[M+H-H_2O]^+$ ion of a generic Δ^7-sterol. The process involves a retro-Diels–Alder reaction initiated from a structure bearing the positive charge localised at C3. The exact *m/z* value, rounded to four decimal places, is reported for the product ion.

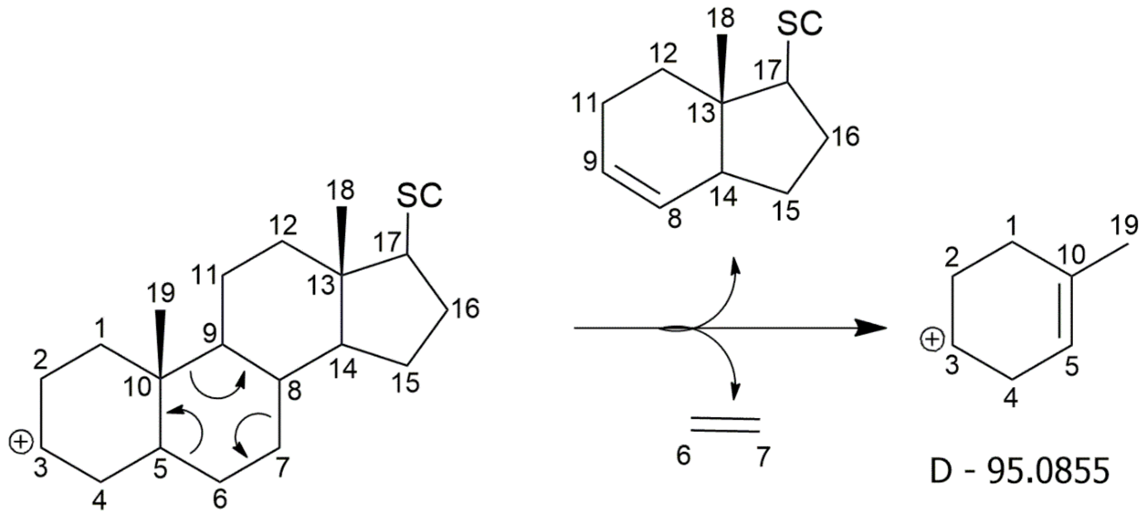

Figure S5. Proposed mechanism for the gas-phase formation of a product ion detected in cluster D and having an exact *m/z* 95.0855 from the $[M+H-H_2O]^+$ ion of a generic stanol. The process involves a retro-cycloaddition on ring B, initiated from a structure bearing the positive charge on C3. The exact *m/z* value, rounded to four decimal places, is reported for the product ion.

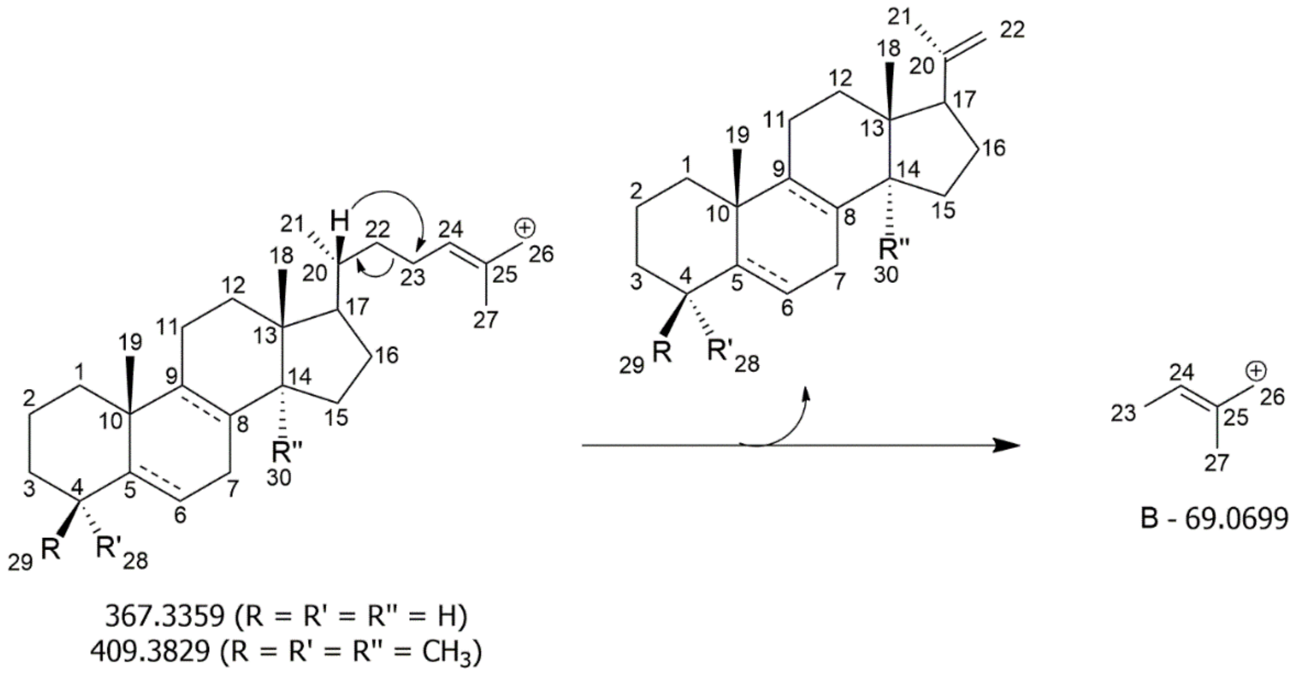

Figure S6. Proposed mechanism for the gas-phase formation of a product ion detected in cluster B in the APCI(+)-HCD-FTMS/MS spectra of the $[M+H-H_2O]^+$ ions of desmosterol and zymosterol (R = R′ = R″ = H; C=C bond at C5–C6 and C8–C9, respectively) and lanosterol (R = R′ = R″ = CH_3; C=C bond at C8–C9). Exact *m/z* ratios are reported to four decimal places.

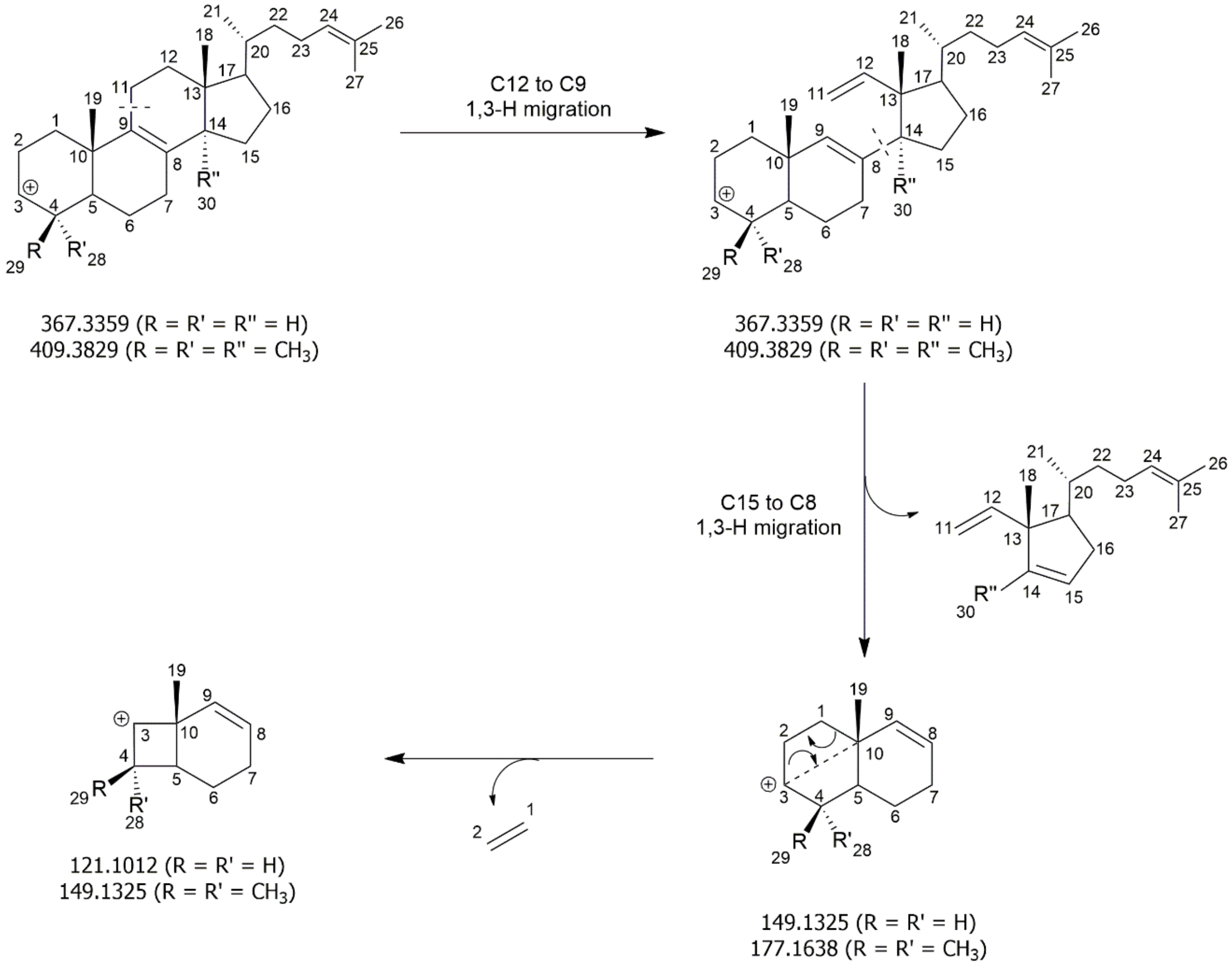

Figure S7. Proposed mechanism for the gas-phase formation of product ions with exact *m/z* values 149.1325/177.1638 and 121.1012/149.1325, corresponding to those observed in the APCI(+)-HCD-FTMS/MS spectra of the $[M+H-H_2O]^+$ ions of zymosterol/lanosterol. Exact *m/z* ratios are reported with four decimal places.

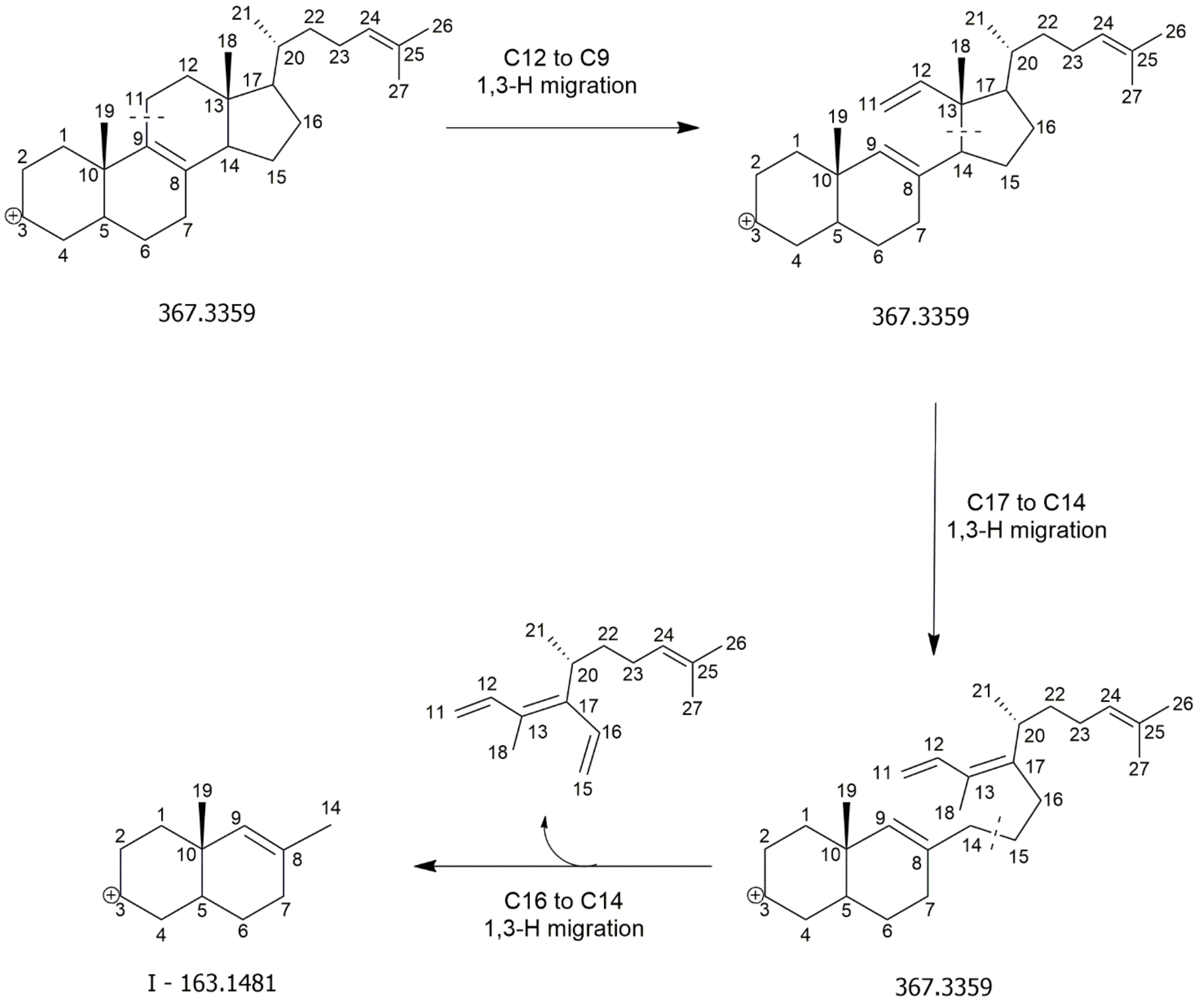

Figure S8. Proposed mechanism for the gas-phase formation of a product ion detected in cluster I in the APCI(+)-HCD-FTMS/MS spectrum of the $[M+H-H_2O]^+$ ion of zymosterol. Exact *m/z* ratios are reported to four decimal places.

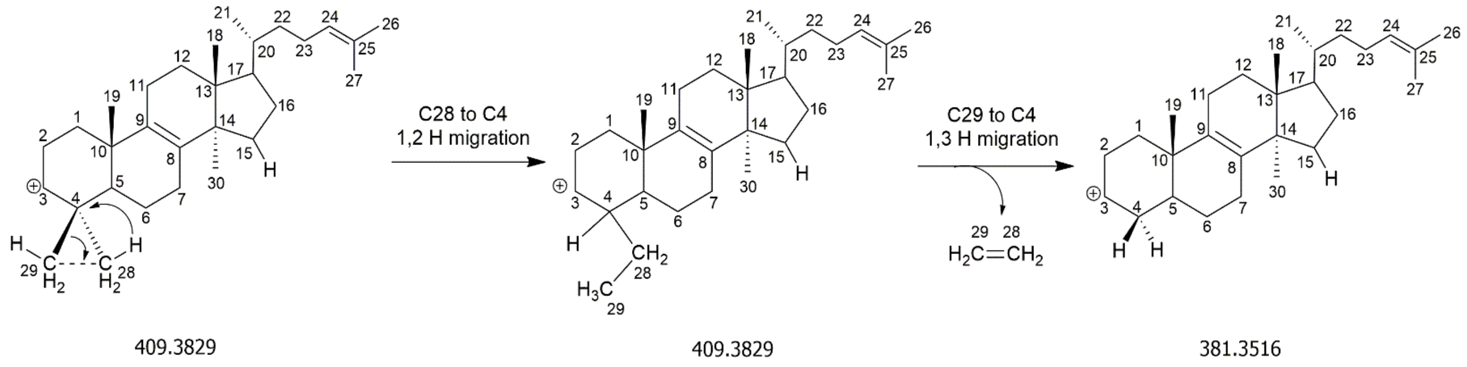

Figure S9. Mechanism tentatively proposed to explain the gas-phase neutral loss of ethylene from the $[M+H-H_2O]^+$ ion of lanosterol, resulting in the replacement of the methyl groups at C4 with hydrogen atoms. Exact *m/z* ratios are reported to four decimal places.

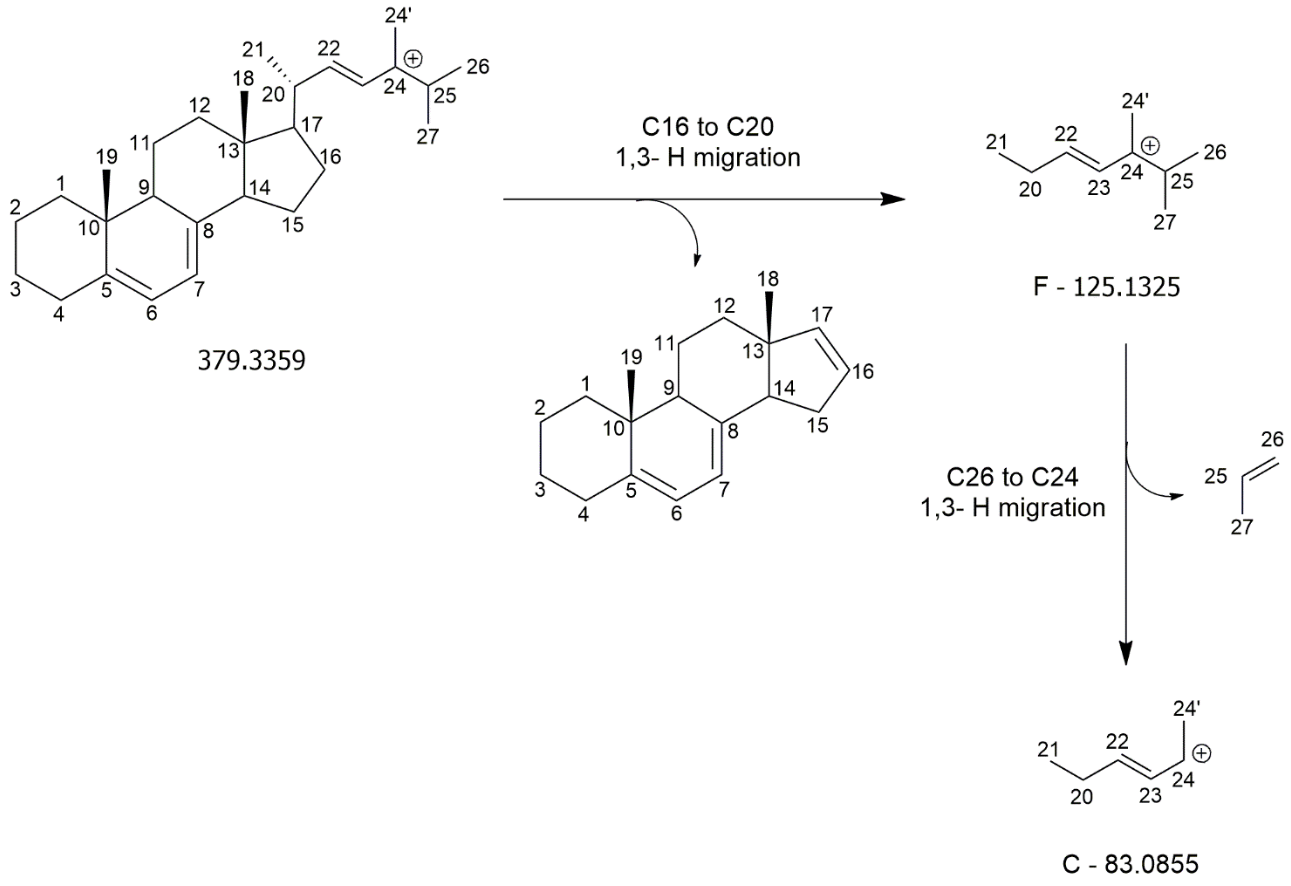

Figure S10. Proposed mechanism for the gas-phase formation of product ions detected in clusters C and F in the APCI-HCD-MS/MS spectrum of the $[M+H-H_2O]^+$ ion of ergosterol. Exact *m/z* ratios are reported to four decimal places.

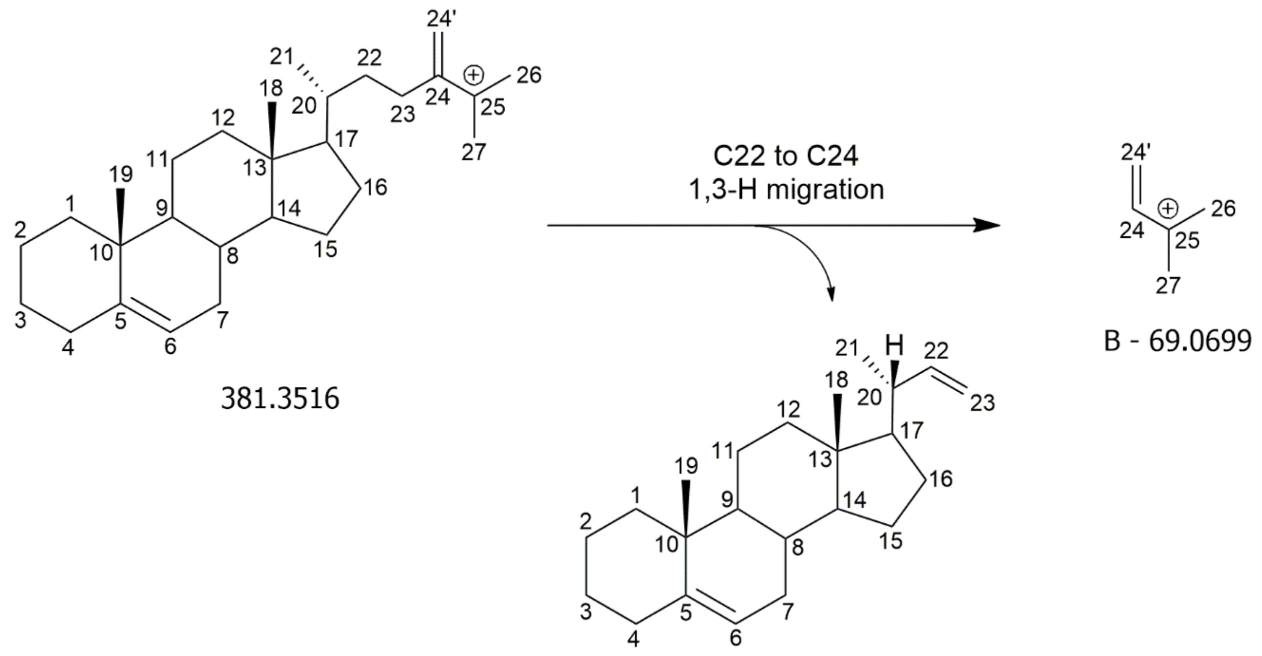

Figure S11. Proposed mechanism for the gas-phase formation of the product ion detected in cluster B in the APCI-HCD-MS/MS spectrum of the $[M+H-H_2O]^+$ ion of chalinasterol. The process involves a 1,3-H transfer from C22 to C24. Exact *m/z* ratios, rounded off to the fourth decimal place, are reported.

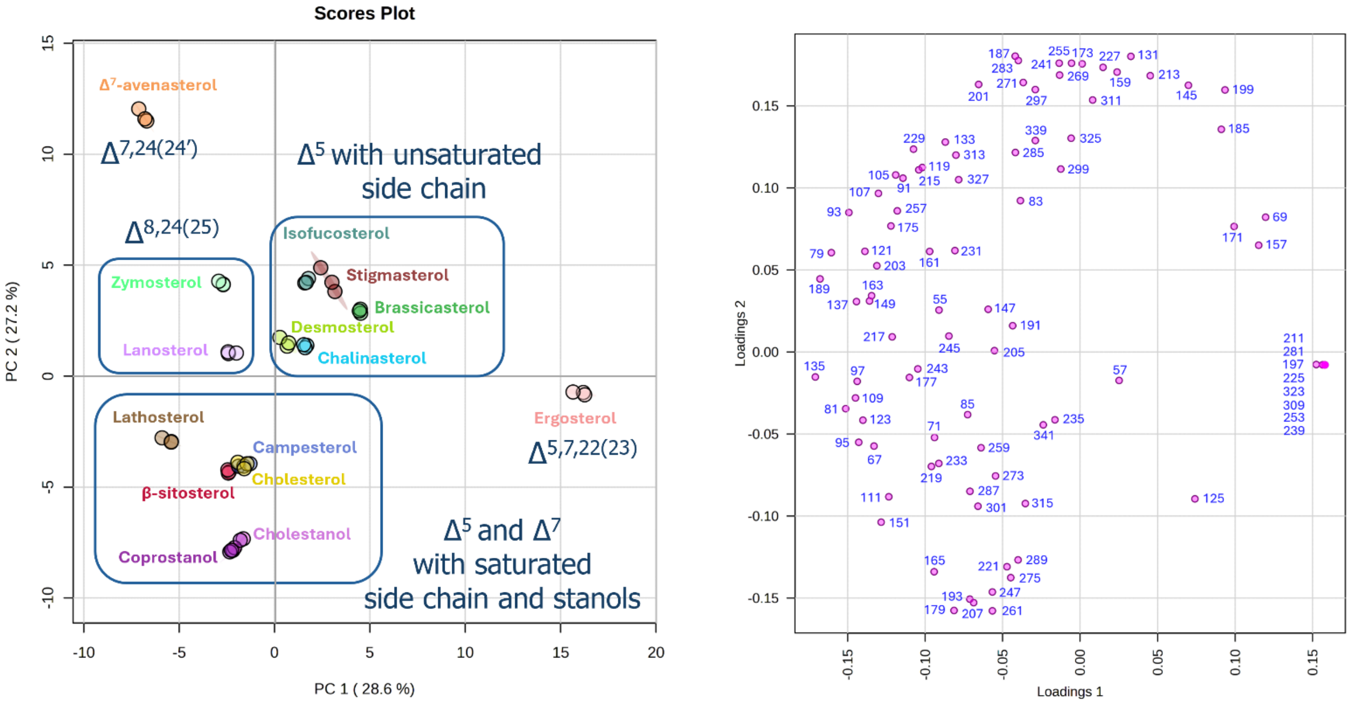

Figure S12. Scores and loading plots obtained from the Principal Component Analysis of relative intensities data referred to product ions detected in APCI(+)-HCD-FTMS/MS spectra of animal/fungal sterols/stanol analyzed in the present study and cholesterol and phytosterols considered in Ref. 49. Each compound was analyzed in triplicate; thus, three symbols are referred to each compound in the scores plot. Major structural characteristics related to groups or single compounds, namely the saturation or unsaturation of the side chain and the occurrence of C=C bonds (Δ) on the steroidal backbone and on the side chain are evidenced. In the loadings plot each point represents the tip of the vector corresponding to a variable, *i.e.*, to a specific product ion, labelled by its nominal *m/z* ratio.

Table S1. Summary of chromatographic resolution and selectivity values evaluated for couples of close sterol/stanol peaks after the RPLC-APCI(+)-FTMS analysis of their standard mixture performed under isocratic or linear gradient conditions (see chromatograms reported in Figure 2 in the main manuscript).

**Compounds**	**Isocratic**		**Linear gradient**	
	**Resolution**	**Selectivity**	**Resolution**	**Selectivity**
Desmosterol - Zymosterol	0.88	1.03	0.00	1.00
Ergosterol - Chalinasterol	0.45	1.02	0.23	1.01
Lathosterol - Cholesterol	0.00	1.00	1.33	1.03
Cholesterol - Lanosterol	1.53	1.06	2.52	1.06
Lanosterol - Coprostanol	3.49	1.13	3.49	1.08
Coprostanol – Cholestanol	5.29	1.19	2.98	1.06

Table S2. Summary of mass spectrometric information concerning product ions detected in the APCI(+)-HCD-FTMS/MS spectra of the $[M+H-H_2O]^+$ ions of sterols/stanols analysed in this study. Product ions are grouped by the number of carbon atoms in their structures, within clusters labelled in alphabetical order, according to Figures 3 and 4. Experimental *m/z* values, expressed with four decimal places, and the corresponding molecular formulas, assigned as those consistent with the best mass accuracy, are reported along with values of the latter. Relative intensities are reported for each ion and each compound, expressed as mean ± standard deviation, as calculated after three independent acquisitions. The predominant relative intensity within each cluster is indicated in bold.

**Ion cluster**	**Molecular formula**	**Experim. *m/z***	**Accuracy [ppm]**	**Relative Intensity [%]**							
				**Latho-sterol**	**Desmo-sterol**	**Zymo-sterol**	**Lano-sterol**	**Chole-stanol**	**Copro-stanol**	**Ergo-sterol**	**Chalina-sterol**
**A**	C_4H_7	55.0551	16	7.8 ± 0.4	1.5 ± 0.4	2.0 ± 0.3	1.9 ± 0.2	1.8 ± 0.2	1.8 ± 0.3	/	3.8 ± 0.2
	C_4H_9	57.0708	16	**14.7 ± 0.6**	**3.8 ± 0.2**	**3.8 ± 0.4**	**5.0 ± 0.2**	**9.4 ± 0.5**	**9.5 ± 0.8**	**14.6 ± 0.6**	**6.8 ± 0.4**
**B**	C_5H_7	67.0552	15	11.0 ± 0.7	6.8 ± 0.4	10.8 ± 0.4	7.0 ± 0.3	14.5 ± 0.7	14.3 ± 0.6	3.7 ± 0.3	5.8 ± 0.2
	C_5H_9	69.0708	13	**23.7 ± 1.4**	**17.6 ± 1.3**	**31 ± 2**	**25.7 ± 0.6**	**19.6 ± 0.5**	**20 ± 0.4**	**100 ± 0**	**11.5 ± 0.4**
	C_5H_{11}	71.0862	10	17 ± 2	3.7 ± 0.5	3.7 ± 0.3	3.1 ± 0.2	9.4 ± 0.5	9.6 ± 0.4	1.7 ± 0.3	4.8 ± 0.3
**C**	C_6H_7	79.0550	10	5.8 ± 0.4	2.9 ± 0.1	4.7 ± 0.4	3.2 ± 0.2	3.6 ± 0.6	3.5 ± 0.4	/	2.0 ± 0.1
	C_6H_9	81.0708	11	56.7 ± 1.5	**37 ± 2**	**59.7 ± 1.4**	**42.0 ± 0.6**	**67.4 ± 1.1**	**68.7 ± 1.1**	9.9 ± 0.2	**29.0 ± 1.4**
	C_6H_{11}	83.0861	7	**59 ± 2**	12.0 ± 1.0	24.6 ± 1.1	18.5 ± 0.9	29.0 ± 1.0	30.8 ± 0.9	**29.1 ± 1.2**	17.5 ± 0.3
	C_6H_{13}	85.1020	9	7.5 ± 1.0	/	/	1.5 ± 0.4	4.4 ± 0.4	4.5 ± 0.5	/	2.2 ± 0.3
**D**	C_7H_7	91.0548	7	4.4 ± 0.9	2.9 ± 0.1	4.9 ± 0.3	1.9 ± 0.1	/	/	/	2.0 ± 0.1
	C_7H_9	93.0706	8	16.5 ± 0.8	10.8 ± 0.2	16.1 ± 0.2	15.8 ± 0.2	9.8 ± 0.4	9.6 ± 0.3	3.1 ± 0.1	7.5 ± 0.7
	C_7H_{11}	95.0863	8	**86.5 ± 1.0**	**44.4 ± 1.3**	**78.5 ± 1.8**	**78.0 ± 1.8**	**100 ± 0**	**100 ± 0**	**9.5 ± 0.4**	**39.0 ± 1.4**
	C_7H_{13}	97.1015	3	34.3 ± 1.2	8.2 ± 0.4	11.7 ± 0.6	14.4 ± 0.7	24.3 ± 0.9	24.9 ± 0.9	2.7 ± 0.3	13.7 ± 0.5
**E**	C_8H_9	105.0701	2	16.6 ± 1.1	9.1 ± 0.3	13.5 ± 0.7	7.4 ± 0.6	/	/	/	6.6 ± 0.3
	C_8H_{11}	107.0858	3	27.5 ± 1.5	17.4 ± 0.4	32.2 ± 0.9	35.8 ± 0.2	16.4 ± 0.7	16.4 ± 0.9	**6.7 ± 0.3**	13.4 ± 0.4
	C_8H_{13}	109.1017	3	**48.3 ± 0.3**	**44.0 ± 1.5**	**71 ± 2**	**95.2 ± 0.9**	**70 ± 2**	**75 ± 2**	6.1 ± 0.1	**36.0 ± 1.4**
	C_8H_{15}	111.1170	2	23.5 ± 0.7	8.9 ± 0.4	8.7 ± 0.4	9.2 ± 0.7	18.0 ± 1.2	17 ± 2	6.3 ± 1.0	6.9 ± 0.1
**F**	C_9H_{11}	119.086	4	16.8 ± 0.6	8.7 ± 0.7	15.8 ± 1.1	11.5 ± 0.7	/	/	3.6 ± 0.4	7.3 ± 0.4
	C_9H_{13}	121.1016	3	29.3 ± 1.4	22.2 ± 0.7	**41.7 ± 1.7**	**51.5 ± 0.9**	29.5 ± 1.6	35.0 ± 0.8	5.8 ± 0.3	19.5 ± 0.7
	C_9H_{15}	123.1172	3	**31 ± 2**	**23.6 ± 0.7**	26.8 ± 0.4	47.4 ± 0.7	**43 ± 2**	**43 ± 2**	6.0 ± 0.1	**21.5 ± 0.7**
	C_9H_{17}	125.1328	2	5.2 ± 0.7	1.7 ± 0.3	/	1.9 ± 0.2	5.1 ± 0.9	5.5 ± 1.0	**9.5 ± 0.5**	3.1 ± 0.2

**G**	$C_{10}H_{11}$	131.0858	2	/	4.2 ± 0.3	4.9 ± 0.4	1.8 ± 0.2	/	/	4.8 ± 0.2	3.2 ± 0.2
	$C_{10}H_{13}$	133.1016	3	20.9 ± 0.2	16.4 ± 0.7	22.4 ± 1.4	14.4 ± 1.3	/	/	**6.8 ± 0.2**	12.5 ± 0.2
	$C_{10}H_{15}$	135.1171	2	**50 ± 3**	**46.3 ± 1.2**	**43 ± 2**	**61.4 ± 1.9**	**55.4 ± 1.0**	**54.7 ± 0.6**	4.1 ± 0.2	**33.5 ± 0.7**
	$C_{10}H_{17}$	137.1327	1	14.8 ± 0.2	21.1 ± 1.0	17.1 ± 0.5	23.3 ± 1.8	21 ± 2	21.3 ± 1.1	/	12.6 ± 0.5
**H**	$C_{11}H_{11}$	143.0853	-1	/	/	/	/	/	/	3.3 ± 0.3	/
	$C_{11}H_{13}$	145.1009	-2	5.3 ± 0.6	8.3 ± 0.5	9.7 ± 0.5	3.5 ± 0.5	/	/	**17.6 ± 0.4**	7.7 ± 0.4
	$C_{11}H_{15}$	147.117	1	39.2 ± 0.2	**59.6 ± 1.1**	30.8 ± 1.5	26.1 ± 1.3	6.4 ± 0.7	6.4 ± 0.7	7.8 ± 0.3	**51 ± 2**
	$C_{11}H_{17}$	149.1328	2	**69 ± 2**	29.3 ± 1.8	**52 ± 2**	**100 ± 0**	**57 ± 2**	**57 ± 2**	5.0 ± 0.1	23.2 ± 0.3
	$C_{11}H_{19}$	151.1484	2	10.9 ± 0.4	5.3 ± 0.4	6.1 ± 0.1	4.6 ± 0.5	14.2 ± 1.6	14.2 ± 1.3	/	6.4 ± 0.3
**I**	$C_{12}H_{13}$	157.1014	1	/	/	/	/	/	/	4.2 ± 0.2	/
	$C_{12}H_{15}$	159.1171	2	6.5 ± 0.5	25.0 ± 1.3	12.9 ± 0.5	5.7 ± 0.3	/	/	**20.0 ± 1.2**	23.5 ± 1.2
	$C_{12}H_{17}$	161.1325	2	**75 ± 2**	**57.5 ± 0.9**	37 ± 2	22.4 ± 1.2	6.0 ± 0.1	6.0 ± 0.2	4.7 ± 0.3	**50.0 ± 1.4**
	$C_{12}H_{19}$	163.1484	2	25.7 ± 0.5	16.1 ± 1.4	**45.4 ± 1.7**	**24.9 ± 0.6**	**32.9 ± 1.8**	**35 ± 2**	3.1 ± 0.1	15.7 ± 0.4
	$C_{12}H_{21}$	165.164	1	4.7 ± 0.6	1.8 ± 0.1	1.8 ± 0.4	1.7 ± 0.3	9.7 ± 0.6	10.1 ± 1.0	/	3.0 ± 0.1
**J**	$C_{13}H_{15}$	171.1169	1	/	/	/	/	/	/	3.2 ± 0.2	/
	$C_{13}H_{17}$	173.1328	2	4.9 ± 0.2	16.2 ± 0.4	11.7 ± 0.4	5.8 ± 0.4	/	/	**5.0 ± 0.1**	15.3 ± 0.5
	$C_{13}H_{19}$	175.1483	1	**43.5 ± 1.3**	**27.1 ± 1.2**	**36.8 ± 1.1**	**13.5 ± 0.8**	6.6 ± 0.7	6.5 ± 0.9	1.9 ± 0.1	**22.6 ± 0.6**
	$C_{13}H_{21}$	177.164	1	8.1 ± 0.4	8.2 ± 0.3	17.6 ± 0.7	10.3 ± 0.3	**21.5 ± 0.7**	**22.0 ± 0.6**	1.8 ± 0.2	10.1 ± 0.2
	$C_{13}H_{23}$	179.1795	1	5.2 ± 0.4	2.1 ± 0.1	1.8 ± 0.3	2.7 ± 0.4	7.2 ± 0.5	7.8 ± 0.7	/	/
**K**	$C_{14}H_{17}$	185.1321	-2	/	1.9 ± 0.3	/	1.8 ± 0.3	/	/	**4.2 ± 0.3**	/
	$C_{14}H_{19}$	187.1483	1	7.7 ± 0.6	10.2 ± 0.2	13.8 ± 0.3	5.9 ± 0.5	/	/	3.2 ± 0.2	10.7 ± 0.4
	$C_{14}H_{21}$	189.1639	1	**29.6 ± 0.5**	**13.9 ± 0.8**	**26.7 ± 0.5**	16.3 ± 0.7	**16.9 ± 1.8**	**17.1 ± 1.8**	/	**12.9 ± 1.3**
	$C_{14}H_{23}$	191.1796	1	6.1 ± 0.2	5.9 ± 0.3	23 ± 0.4	78.1 ± 1.7	8.3 ± 0.4	8.5 ± 0.4	/	5.8 ± 0.3
	$C_{14}H_{25}$	193.1955	2	7.3 ± 0.3	1.9 ± 0.1	/	/	7.1 ± 0.5	7.6 ± 0.5	/	/
**L**	$C_{15}H_{17}$	197.1321	-2	/	/	/	/	/	/	2.0 ± 0.1	/
	$C_{15}H_{19}$	199.1483	1	/	4.8 ± 0.4	3.8 ± 0.5	1.8 ± 0.2	/	/	**6.7 ± 0.2**	5.8 ± 0.3
	$C_{15}H_{21}$	201.1639	0	16.7 ± 0.7	11.5 ± 1.1	29.2 ± 0.4	7 ± 0.1	/	/	4.2 ± 0.3	12.5 ± 0.7
	$C_{15}H_{23}$	203.1797	1	**33.7 ± 0.6**	**19.7 ± 0.9**	**41.5 ± 1.5**	**47.8 ± 1.0**	**14.7 ± 0.6**	**16.4 ± 1.7**	/	**20.0 ± 0.5**
	$C_{15}H_{25}$	205.1954	1	12.8 ± 0.5	6.4 ± 0.4	9.1 ± 0.5	45 ± 2	6.9 ± 0.7	6.4 ± 1.2	/	/

	$C_{15}H_{27}$	207.2109	1	7.8 ± 0.4	/	/	/	9.0 ± 0.9	9.9 ± 0.1	/	/
**M**	$C_{16}H_{19}$	211.1477	-2	/	/	/	/	/	/	2.1 ± 0.1	/
	$C_{16}H_{21}$	213.164	1	/	6.7 ± 0.6	5.8 ± 0.4	1.7 ± 0.3	/	/	**5.0 ± 0.1**	7.4 ± 0.5
	$C_{16}H_{23}$	215.1796	1	**45.9 ± 1.6**	**18.8 ± 1.1**	**41 ± 2**	6.6 ± 0.4	/	/	2.0 ± 0.1	**16.0 ± 0.1**
	$C_{16}H_{25}$	217.1952	0	16.5 ± 1.3	9.4 ± 0.6	17.8 ± 0.7	**32.6 ± 0.6**	**9.9 ± 0.8**	**9.9 ± 0.8**	/	7.3 ± 0.5
	$C_{16}H_{27}$	219.211	1	12.0 ± 0.6	4.1 ± 0.2	/	5.6 ± 0.6	5.6 ± 0.6	5.7 ± 0.6	/	3.9 ± 0.1
	$C_{16}H_{29}$	221.2257	-3	/	/	/	/	1.8 ± 0.2	1.8 ± 0.3	/	/
**N**	$C_{17}H_{21}$	225.1631	-3	/	/	/	/	/	/	2.2 ± 0.2	/
	$C_{17}H_{23}$	227.1789	-2	/	4.0 ± 0.2	3.9 ± 0.4	/	/	/	**2.7 ± 0.3**	3.3 ± 0.5
	$C_{17}H_{25}$	229.1946	0	**14.8 ± 0.8**	**13.1 ± 1.1**	**16.1 ± 0.3**	6.7 ± 0.3	/	/	/	**11.7 ± 1.0**
	$C_{17}H_{27}$	231.211	1	3.7 ± 0.7	8.1 ± 0.4	9.1 ± 0.5	**8.4 ± 0.8**	**6.3 ± 0.3**	**5.5 ± 0.3**	/	5.9 ± 0.2
	$C_{17}H_{29}$	233.2266	1	6.3 ± 0.8	4.5 ± 0.6	/	/	5.2 ± 0.2	5.4 ± 0.4	/	2.5 ± 0.8
	$C_{17}H_{31}$	235.2421	0	/	/	/	/	/	/	/	/
**O**	$C_{18}H_{23}$	239.179	-2	/	/	/	/	/	/	**3.8 ± 0.2**	/
	$C_{18}H_{25}$	241.1953	1	/	5.2 ± 0.4	3.8 ± 0.3	3.9 ± 0.2	/	/	1.6 ± 0.3	5.8 ± 0.3
	$C_{18}H_{27}$	243.2102	-2	**21.4 ± 0.6**	**12.3 ± 0.3**	**10.5 ± 1.1**	**12.2 ± 0.2**	/	/	/	**6.7 ± 0.5**
	$C_{18}H_{29}$	245.2258	-2	6.0 ± 0.2	6.0 ± 0.1	**1.9 ± 0.1**	4.3 ± 0.6	4.5 ± 0.5	4.5 ± 0.3	/	6.2 ± 0.2
	$C_{18}H_{31}$	247.2414	-2	5.7 ± 0.6	/	/	/	**7.0 ± 0.1**	**6.5 ± 0.4**	/	3.3 ± 0.5
**P**	$C_{19}H_{25}$	253.1952	0	/	/	/	/	/	/	**9.8 ± 0.3**	/
	$C_{19}H_{27}$	255.2108	0	2.7 ± 0.5	8.0 ± 0.3	10.1 ± 0.3	3.9 ± 0.2	/	/	1.8 ± 0.2	**9.6 ± 0.5**
	$C_{19}H_{29}$	257.2265	0	**26.0 ± 0.4**	**15.6 ± 1**	**28 ± 1.0**	**11.5 ± 0.7**	/	/	/	9.3 ± 0.4
	$C_{19}H_{31}$	259.2422	1	17.7 ± 1.2	4.1 ± 0.2	/	3.2 ± 0.3	3.6 ± 0.5	3.4 ± 0.4	/	3.0 ± 0.2
	$C_{19}H_{33}$	261.2581	2	1.8 ± 0.4	/	/	/	**10.1 ± 0.6**	**10.2 ± 0.2**	/	/
**Q**	$C_{20}H_{27}$	267.2102	-2	/	/	/	/	/	/	**1.7 ± 0.3**	/
	$C_{20}H_{29}$	269.2257	-3	/	1.8 ± 0.4	6.9 ± 0.4	2.0 ± 0.2	/	/	/	2.3 ± 0.4
	$C_{20}H_{31}$	271.242	0	/	**7.1 ± 0.2**	**7.8 ± 0.5**	**6.4 ± 0.5**	/	/	/	**8.7 ± 0.4**
	$C_{20}H_{33}$	273.2577	0	**10.7 ± 0.6**	1.5 ± 0.3	**1.7 ± 0.4**	1.9 ± 0.1	2.2 ± 0.2	2.2 ± 0.2	/	3.8 ± 0.4
	$C_{20}H_{35}$	275.2735	1	/	/	/	/	**5.8 ± 1.2**	**5.8 ± 1.2**	/	/
**R**	$C_{21}H_{29}$	281.226	-1	/	/	/	/	/	/	**1.7 ± 0.1**	/
	$C_{21}H_{31}$	283.2424	1	/	4.5 ± 0.5	9.2 ± 0.5	1.6 ± 0.4	/	/	/	**4.9 ± 0.2**

	$C_{21}H_{33}$	285.2579	1	1.9 ± 0.4	**9.5 ± 0.8**	**26 ± 2**	**4.1 ± 0.2**	/	/	/	4.3 ± 0.4
	$C_{21}H_{35}$	287.2736	1	**24.9 ± 1.9**	/	/	/	2.1 ± 0.1	2.2 ± 0.2	/	/
	$C_{21}H_{37}$	289.2892	1	/	/	/	/	**3.1 ± 0.1**	**3.0 ± 0.2**	/	/
**S**	$C_{22}H_{31}$	295.2418	-1	/	/	/	/	/	/	**4.2 ± 0.2**	/
	$C_{22}H_{33}$	297.258	1	/	**3.0 ± 0.1**	**5.8 ± 0.4**	3.1 ± 0.2	/	/	/	**8.1 ± 0.1**
	$C_{22}H_{35}$	299.2734	0	/	/	3.2 ± 0.2	**5.5 ± 1.0**	/	/	/	5.9 ± 0.2
	$C_{22}H_{37}$	301.2891	0	**9.7 ± 0.4**	/	/	/	**2.1 ± 0.1**	**2.1 ± 0.1**	/	/
**T**	$C_{23}H_{33}$	309.2579	1	/	/	/	/	/	/	**4.9 ± 0.1**	/
	$C_{23}H_{35}$	311.2734	0	/	**3.4 ± 0.4**	**12.1 ± 0.5**	**5.7 ± 0.6**	/	/	/	**7.9 ± 0.3**
	$C_{23}H_{37}$	313.289	0	**3.2 ± 0.5**	/	/	1.8 ± 0.1	/	/	/	/
	$C_{23}H_{39}$	315.3047	0	/	/	/	/	**3.1 ± 0.2**	**2.9 ± 0.1**	/	/
**U**	$C_{24}H_{35}$	323.2727	-2	/	/	/	/	/	/	**1.5 ± 0.3**	/
	$C_{24}H_{37}$	325.2892	1	/	/	/	**3.7 ± 0.2**	/	/	/	**1.9 ± 0.1**
	$C_{24}H_{39}$	327.3047	0	/	/	/	2.1 ± 0.3	/	/	/	/
**V**	$C_{25}H_{39}$	339.3049	1	/	/	/	2.6 ± 0.5	/	/	/	/
	$C_{25}H_{41}$	341.3205	1	/	/	/	/	/	/	/	/
**Precursor ion**	$C_{27}H_{43}$	367.3362	1	/	/	**100 ± 0**	/	/	/	/	/
	$C_{27}H_{45}$	369.3519	1	**100 ± 0**	**100 ± 0**	/	/	/	/	/	/
	$C_{27}H_{47}$	371.3672	1	/	/	/	/	**42.7 ± 2.1**	**42.7 ± 2.1**	/	/
	$C_{28}H_{43}$	379.3363	1	/	/	/	/	/	/	**16.2 ± 1.3**	/
	$C2_{8}H_{45}$	381.3519	1	/	/	/	/	/	/	/	**100 ± 0**
	$C_{30}H_{49}$	409.3832	1	/	/	/	**33.4 ± 1.1**	/	/	/	/
